# Supplementary material for: New Structures, Spectrometric Quantification, and Inhibitory Properties of Cardenolides from Asclepias curassavica Seeds
Source: Molecules. 2022 Dec 23;28(1):105. doi: 10.3390/molecules28010105 (PMC9822358; doi:10.3390/molecules28010105)
Supplement: Supplementary file 1 [file molecules-28-00105-s001.zip › molecules-2070226-supplementary.pdf]

Supporting Information

New structures, spectrometric quantification and Na^+/K^+ inhibitory properties from cardenolides of *Asclepias curassavica* seeds

Authors

Paola A. Rubiano-Buitrago[1,2], Shrikant Pradhan[1], Christian Paetz[2] and Hannah M. Rowland[1]

Affiliations

1. Research Group Predators and toxic prey, Max Planck Institute for Chemical Ecology, Jena, Germany, pbuitrago@ice.mpg.de (PARB), hrowland@ice.mpg.de (HMR).
2. Research Group Biosynthesis/NMR, Max Planck Institute for Chemical Ecology, Jena, Germany, cpaetz@ice.mpg.de.

Correspondence: PRB pbuitrago@ice.mpg.de HMR hrowland@ice.mpg.de

Contents

No.	Content	Page
1	**Figure S1.** UV and MS chromatograms of *Asclepias curassavica* extract	S4
2	**Figure S2.** Calibration curves of compounds **1-10**	S4
3	**Table S1**. ^{1}H and ^{13}C NMR data compounds **5-10**	S5-6
4	**Figure S3:** HR-ESI-MS spectrum of compound **1.**	S7
5	**Figure S4:** UV spectrum of compound **1**	S8
6	**Figure S5:** Compound **1**-NMR shift assignment	S8
7	**Figure S6.** ^{1}H-NMR spectrum (500 MHz, D_2O) of compound **1**.	S9
8	**Figure S7.** DEPTQ spectrum (125 MHz, D_2O) of compound **1**.	S9
9	**Figure S8.** HSQC spectrum (500 MHz, D_2O) of compound **1.**	S10
10	**Figure S9.** ^{1}H-^{1}H COSY spectrum (500 MHz, D_2O) of compound **1.**	S10
11	**Figure S10.** ^{1}H-^{13}C HMBC spectrum (500 MHz, D_2O) of compound **1.**	S11
12	**Figure S11.** ROESY spectrum (500 MHz, D_2O) of compound **1.**	S11
13	**Figure S12.** HSQC-TOCSY spectrum (500 MHz, D_2O) of compound **1.**	S12
14	**Figure S13.** SELTOCSY spectrum (500 MHz, transmitter frequency at 3.609 ppm (H-6′), D_2O) of compound **1.**	S12
15	**Figure S14.** SELTOCSY spectrum (500 MHz, transmitter frequency at 3.765 ppm (H-3), D_2O) of compound **1.**	S13
16	**Figure S15:** HR-ESI-MS spectrum of compound **2.**	S14
17	**Figure S16.** UV spectrum of compound **2.**	S15
18	**Figure S17:** Compound **2**-NMR shift assignment	S15
19	**Figure S18.** ^{1}H-NMR spectrum (500 MHz, D_2O) of compound **2**.	S15
20	**Figure S19.** DEPTQ spectrum (125 MHz, D_2O) of compound **2**.	S16
21	**Figure S20.** HSQC spectrum (500 MHz, D_2O) of compound **2.**	S17
22	**Figure S21.** ^{1}H-^{1}H COSY spectrum (500 MHz, D_2O) of compound **2.**	S17
23	**Figure S22.** ^{1}H-^{13}C HMBC spectrum (500 MHz, D_2O) of compound **2.**	S18
24	**Figure S23.** ROESY spectrum (500 MHz, D_2O) of compound **2.**	S18
25	**Figure S24.** HSQC-TOCSY spectrum (500 MHz, D_2O) of compound **2.**	S19
26	**Figure S25.** SELTOCSY spectrum (500 MHz, transmitter frequency at 4.87 ppm (H-1′), D_2O) of compound **2.**	S19
27	**Figure S26.** SELTOCSY spectrum (500 MHz, transmitter frequency at 4.45 ppm (H-1′′), D_2O) of compound **2.**	S20
28	**Figure S27:** HR-ESI-MS spectrum of compound **3.**	S21
29	**Figure S28:** UV spectrum of compound **3.**	S22
30	**Figure S29:** Compound **3**-NMR shift assignment	S22
31	**Figure S30.** ^{1}H-NMR spectrum (500 MHz, D_2O) of compound **3**.	S23
32	**Figure S31.** DEPTQ spectrum (125 MHz, D_2O) of compound **3**.	S23

33 **Figure S32.** HSQC spectrum (500 MHz, D_2O) of compound **3.** S24

34 **Figure S33.** ^{1}H-^{1}H COSY spectrum (500 MHz, D_2O) of compound **3.** S24

35 **Figure S34.** ^{1}H-^{13}C HMBC spectrum (500 MHz, D_2O) of compound **3.** S25

36 **Figure S35.** ROESY spectrum (500 MHz, D_2O) of compound **3.** S25

37 **Figure S36.** HSQC-TOCSY spectrum (500 MHz, D_2O) of compound **3.** S26

38 **Figure S37:** HR-ESI-MS spectrum of compound **4.** S27

39 **Figure S38:** UV spectrum of compound **4.** S28

40 **Figure S39:** Compound **4**-NMR shift assignment S28

41 **Figure S40.** ^{1}H-NMR spectrum (500 MHz, D_2O) of compound **4**. S29

42 **Figure S41.** APT spectrum (125 MHz, D_2O) of compound **4**. S29

43 **Figure S42.** HSQC spectrum (500 MHz, D_2O) of compound **4.** S30

44 **Figure S43.** ^{1}H-^{1}H COSY spectrum (500 MHz, D_2O) of compound **4.** S30

45 **Figure S44.** ^{1}H-^{13}C HMBC spectrum (500 MHz, D_2O) of compound **4.** S31

46 **Figure S45.** ROESY spectrum (500 MHz, D_2O) of compound **4.** S31

47 **Figure S46.** HSQC-TOCSY spectrum (500 MHz, D_2O) of compound **4.** S32

48 **Figure S47.** SELTOCSY spectrum (500 MHz, transmitter frequency at 4.514 ppm, D_2O) of compound **4.** S32

49 **Figure S48.** SELNOESY spectrum (500 MHz, transmitter frequency at 3.764 ppm (H-3), D_2O) of compound **4.** S33

50 **Figure S49.** SELNOESY spectrum (500 MHz, transmitter frequency at 3.223 ppm (H-17), D_2O) of compound **4.** S33

51 **Table S2:** Cardenolides quantification (mg/g of seeds) S34

52 **Figure S50:** Inhibition curves of *Sus domesticus* Na^+/K^+ ATPase by compounds **1**,**2**, **5**—**10** and ouabain S34

53 **Table S3:** IC_{50} values (μM) of compounds **1**,**2**,**5**—**10** and ouabain against porcine Na^+/K^+ ATPase. S35

54 **Table S4:** Comparison of IC_{50} values between compounds **1**,**2**,**5**—**10** along with ouabain, p-values adjusted (* $p < 0.05$; ** $p < 0.01$, *** $p < 0.001$). S35

Figure S1: UV and MS chromatograms of *Asclepias curassavica* extract (compounds **1-10** above their correspondent peak)

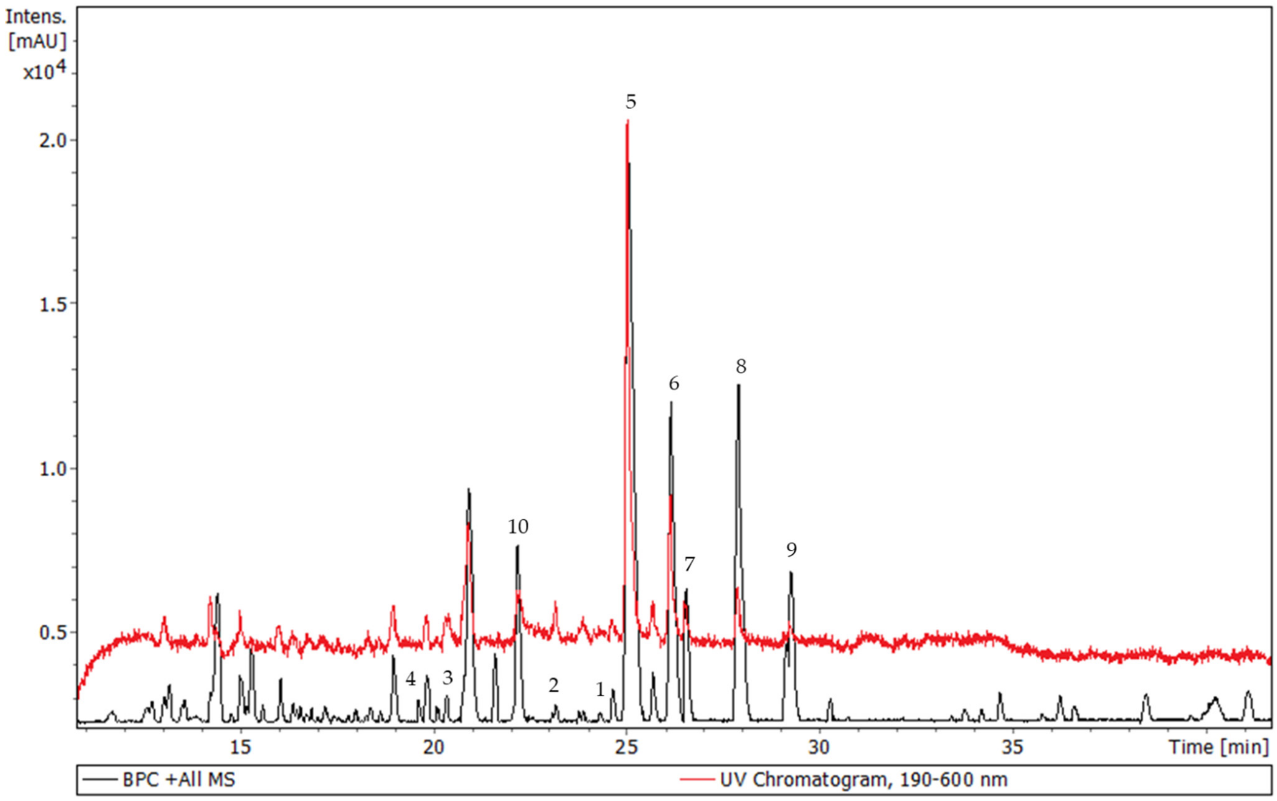

Figure S2: Calibration curves of compounds 1-10

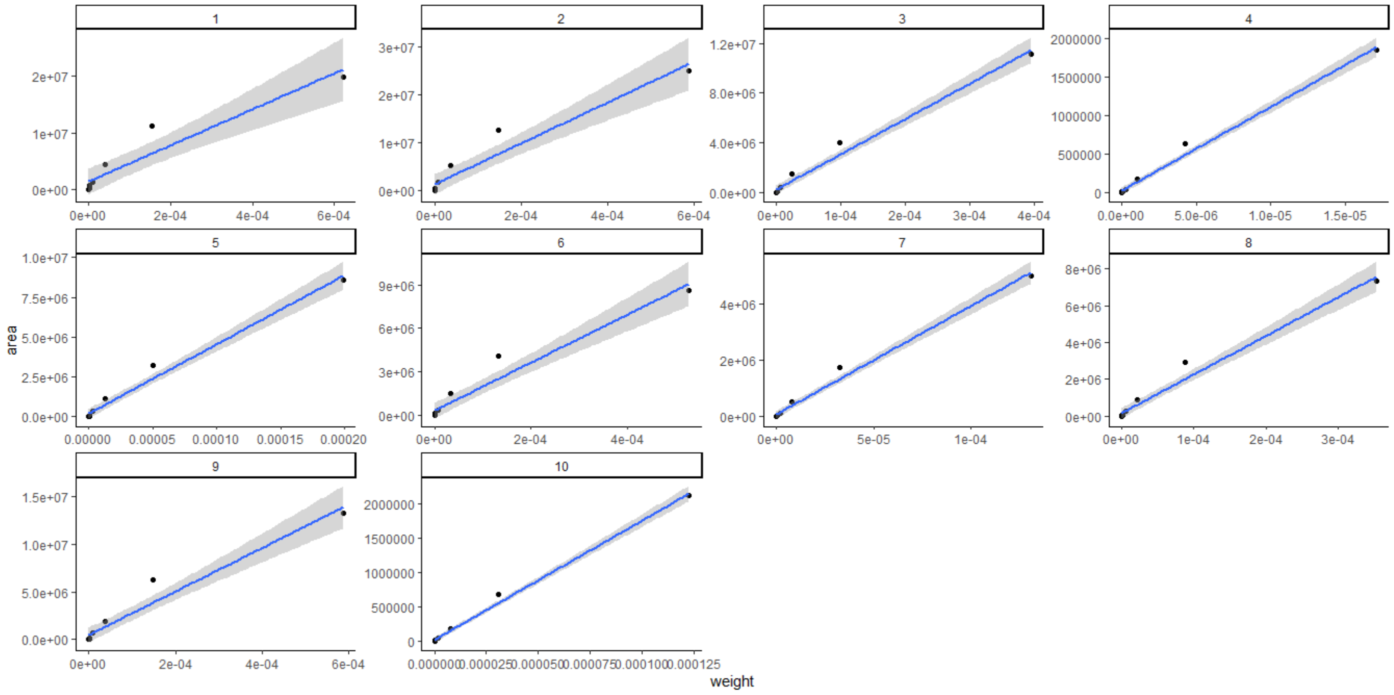

Table S1: ^{1}H NMR (500 MHz) and ^{13}C (125 MHz) data of compounds **5-10** [δ in ppm, configuration, multiplicity (*J* in HZ)]

Position	**5***		**6***		**7***		**8****		**9***		**10***	
	δ_H	δ_C	δ_H	δ_C	δ_H	δ_C	δ_H	δ_C	δ_H	δ_C	δ_H	δ_C
1	2.26 β, 0.92 α	30.8	2.39 bd (13.4) 1.06 bt (13.4)	30.1	2.46 β dd (12.6,4.3) 1,19 α t (12.6)	34.4	2.39 β dt (13.6,2.9) 1.08 bt (13.6)	30	2.31 β dt (13.1, 3.3) 0.78 td (13.1, 3.3)	32.5	2.37 β dd (12.6,4.4) 1.12 α t (12.6)	34.3
2	1.44, 1.94	29.1	1.97, 1.24	29.8	3.90 β	69.1	1.98, 1.24	29.8	1.88, 1.53	30.7	3.74 β ddd (12.6,10.7, 4.4)	69
3	3.85 α	78.8	3.79	78.4	4.03 α t (3.0)	72	3.83 α	78.3	3.67 α	79.2	3.89 α td (10.7, 4.0)	72.1
4	1.41, 1.83	34	1.99, 1.21	35.4	1.78, 1.35 β	30.8	1.99 α, 1.2 β	35.7	1.76, 1.42	35.8	1.67, 1.24 q (12.6)	32.7
5	1.3 α	43.8	1.51	42.1	1.71	42.3	1.6 td (12.2, 3.1)	42.3	1.21	45.6	1.6 α	42.3
6	1.36, 1.36	26.7	1.94, 1.68	27.7	2.19, 1.77	26.7	2.16, 1.35	26.7	1.31, 1.21	29.3	1.86 bdd (13.1, 3.3), 1.26	26.8
7	1.98, 1.18	27.1	2.16 bd (12.3) 1.32	26.8	1.98 β qd (13,1,3.1) 1.39	26.8	1.95 β, 1.69	27.8	2.06, 1.13	28.6	2.08, 1.66	26.7
8	1.74 β	41.3	1.6 t (12.4)	42.3	1.65β	41.6	1.53 β	42	1.77	42.8	1.53 β	41.6
9	1.12 α	49.2	1.41	47.8	1.58	47.5	1.44 α	47.7	1 α m	51.3	1.52 α	47.4
10		39.1		51.9		53.2		52.1		40.3		53.2
11	1.66, 1.44	22.5	1.71, 1.12	21.5	1.73 1.16 β	21.5	1.73, 1.13 β	21.5	1.62, 1.62	23.7	1.68, 1.09	21.8
12	1.44, 1.55 α	39.7	1.53, 1.44	38.7	1.56, 1.47, bt (13.6)	38.4	1.52, 1.44	38.9	1.48, 1.39	41.3	1.63, 1.57	39.6
13		49.2		49.4		49.4		49.5		50.9		48.9
14		86.3		85.7		85.6		85.8		86.4		85.4
15	2.20, 1.73	31.7	2.2, 1.71	31	2.16, 1.74	30.8	2.14, 1.71	31.1	2.14, 1.69 dd (11.5;9.5)	33.3	2.06, 2.06	39.1
16	2.22, 1.84	26.5	2.12, 1.83	26.5	2.21, 1.82	26.4	2.21, 1.83	26.5	2.16, 1.86	27.8	4.48 β td (8.2,4.1)	76.1
17	2.91 α	50.1	2.88	49.9	2.92 dd (9.5, 5.6)	49.7	2.9 α dd (9.5, 5.4)	50.2	2.82 α	52.1	2.62 α d (5.1)	59.2
18	0.95 s β	15.2	0.81 β s	15.1	0.83 β s	14.9	0.82 β s	15.1	0.92 β s	16.2	0.71 β s	14.9
19	3.92, 3.77	60.1	10 s	214.9	10.09 β s	212.8	9.95 β s	214.9	3.86 dd (11.7, 4.2) 3.73	60	9.96 s	212.9
20		178.9		178.7		178.4		178.5		176.9		175.4
21	5.06 dd (18.2, 1.3) 5.08 dd (18.2, 1.3)	75	5.06 bd (18.5) 5.01 bd (18.5)	75	5.07 dd (18.2, 1.3), 5.04 dd (18.2, 1.3)	75	5.08, 5.03	75.1	5.03, 4.90	75.2	4.92, 4.92	75.1
22	6.00 s	116.1	5.98 s	115.8	6.00 s	116	5.97 s	116	5.89 s	117.5	5.96 s	116.7
23		179.4		178.9		178.8		178.9		178.1		177.9
1´	4.87α d (8.3)	97.9	4.82 α d (7.9)	97.8	4.65 β s	94.8	4.81α d (8.2)	98	4.72 d (8.0)	99.5	4.53 β s	95.2
2´	3.46 β	69.7	3.4 β dd (7.7,1.8)	70		91.6	3.38 dd (8.2,3.1)	70.5	3.26 dd (7.9, 2.9)	72.6		91.8

3´	4.4 β t (2.8)	70.7	4.37 β bd	70.5	3.96 β dd (11.6, 4.5) 2.15 β bdd (12.0, 4.5)	81.5	4.13 α t (3.1)	71.2	4.01 α t (2.9)	73	3.68 β dd (12.1,5.0) 1.76 β ddd (12.6,4.8,1.3),	71.6
4´	3.53 β	82	3.49β d (9.4)	81.6	1.73 q (11.3, 12.0)	36.9	3.35 dd(9.8,3.1)	72.4	3.14 dd (9.5,2.7)	74.6	1.54 α	37.2
5´	3.94 α	68.2	3.9 α bdt (6.2, 6.2, 3.2)	68.4	3.84 β dd (11.3,5.9)	68.6	3.8 α m	69.4	3.71 α	70.4	3.72 β	68.9
6´	1.34 β d (6.2)	16.8	1.31 β d (6.2)	16.8	1.3 α d (5.9)	19.7	1.25 β d (5.9)	16.7	1.22 β d (6.3)	18	1.19 α d (5.7)	19.7
1"	4.6 α d (7.8)	104	4.58 α d (7.8)	104	4.66 α d (7.9)	104						
2"	3.35 β bt (8.3)	73.1	3.32 β bt (8.8)	73.2	3.39 bt (8.7)	73.3						
3"	3.5 α	75.7	3.43 α	75.6	3.53 α t (9.1)	75.5						
4"	3.46 β	75.7	3.42 β	69.4	3.44 m	69.3						
5"	3.46 α	75.8	3.48 α t (9.4)	75.6	3.46 α m	75.8						
6"	3.92, 3.77	60.1	3.89 bd (12.1) 3.75 dd (12.1,3.3)	60.2	3.92 bd (12.2) 3.76 dd (12.2, 5.1)	60.4						

*-in D_2O, **-in MeOD-d3

Figure S3: HR-ESI-MS spectrum of compound **1.**

Compound Spectrum List Report

Analysis Info

		Acquisition Date	08/03/2022 11:25:26
Analysis Name	\\filer\bird-lab\Users\Paola R-B\LCMS data\Asclepias extracts\553 calibration\allosyl-corog 8.3.22 dilu 2_P1-E-2_01_172.d		
Method	paola_lcms_pos_checkuplc040221.m	Operator	MPI Jena
Sample Name	allosyl-corog 8.3.22 dilu 2	Instrument	compact 8255754.20124
Comment			

Acquisition Parameter

Source Type	ESI	Ion Polarity	Positive	Set Nebulizer	1.8 Bar
Focus	Not active	Set Capillary	4500 V	Set Dry Heater	220 °C
Scan Begin	50 m/z	Set End Plate Offset	-500 V	Set Dry Gas	9.0 l/min
Scan End	1300 m/z	Set Charging Voltage	2000 V	Set Divert Valve	Waste
		Set Corona	0 nA	Set APCI Heater	0 °C

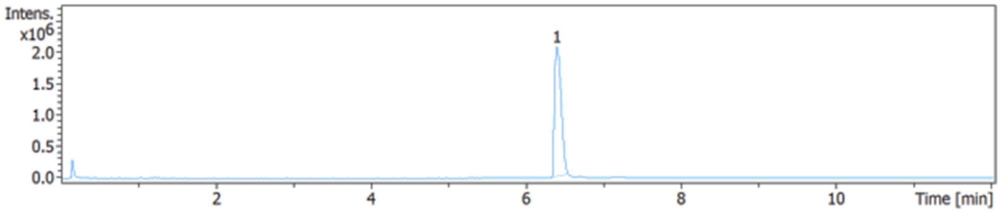

#	RT [min]	Area	Int. Type	I	S/N	Trace	Max. m/z	FWHM [min]
1	6.4	12135308	Manual	2092461	3447.7	EIC 553.3017 +All MS	553.3015	0.1

Cmpd 1, 6.4 min

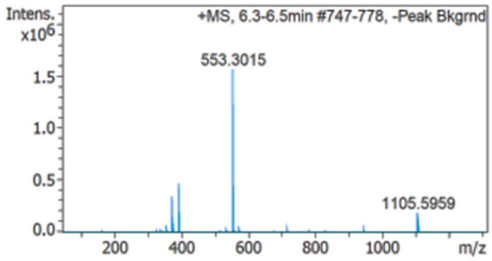

#	m/z	Res.	S/N	I	I %	FWHM
1	373.2377	11047	8971.6	347780	22.2	0.0338
2	374.2419	11063	2629.5	102053	6.5	0.0338
3	391.2483	11304	11511.1	468366	29.9	0.0346
4	392.2521	11232	3403.2	138660	8.9	0.0349
5	553.3015	11452	20743.1	1565642	100.0	0.0483
6	554.3050	11599	7294.6	551036	35.2	0.0478
7	555.3088	11604	1947.4	147316	9.4	0.0479
8	943.5433	12025	866.9	66771	4.3	0.0785
9	1105.5959	11978	3710.1	189855	12.1	0.0923
10	1106.5992	11915	2676.7	136792	8.7	0.0929

Figure S4: UV spectrum of compound **1**

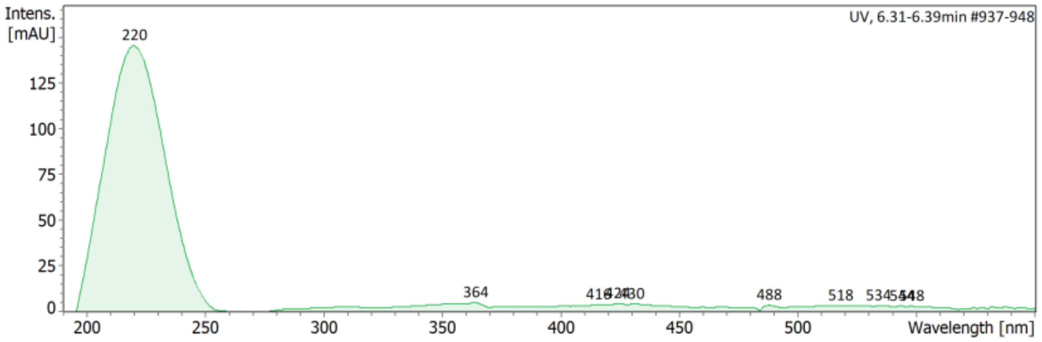

Figure S5: Compound **1**-NMR shift assignment

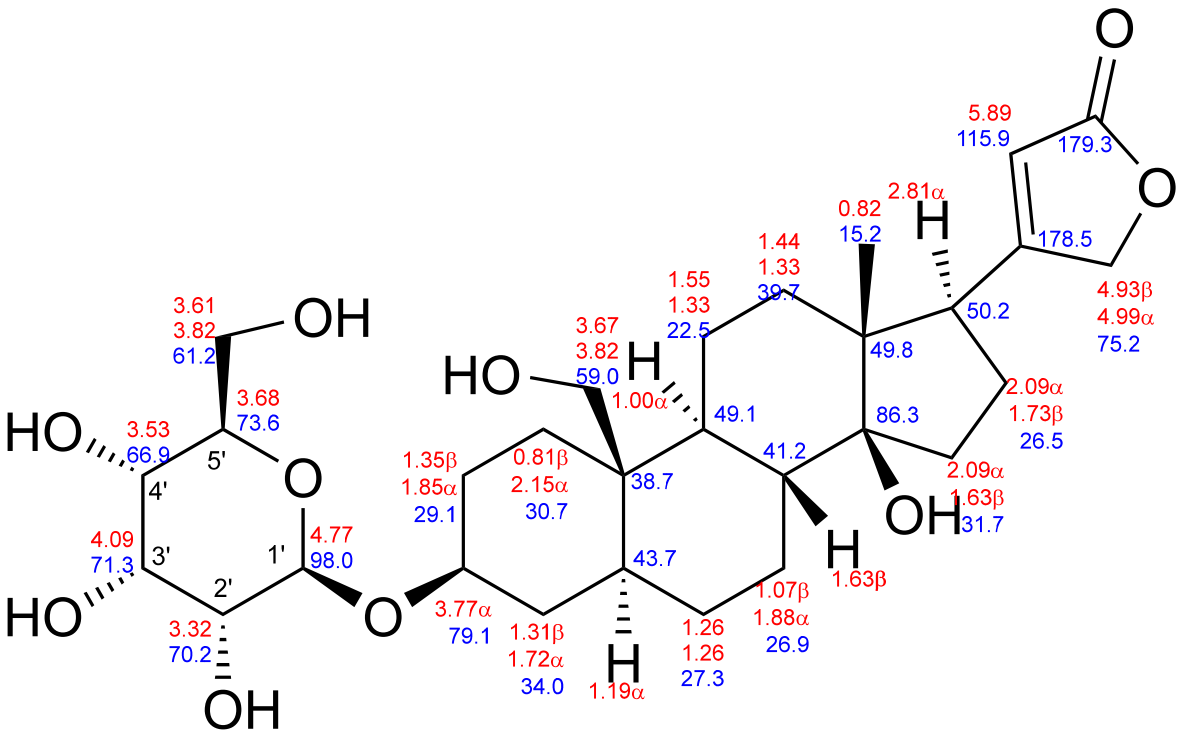

3β-(β-D-allopyranosyloxy)-14,19-dihydroxy-5α-card-20(22)-enolide

Figure S6. ^{1}H-NMR spectrum (500 MHz, D_2O) of compound **1**.

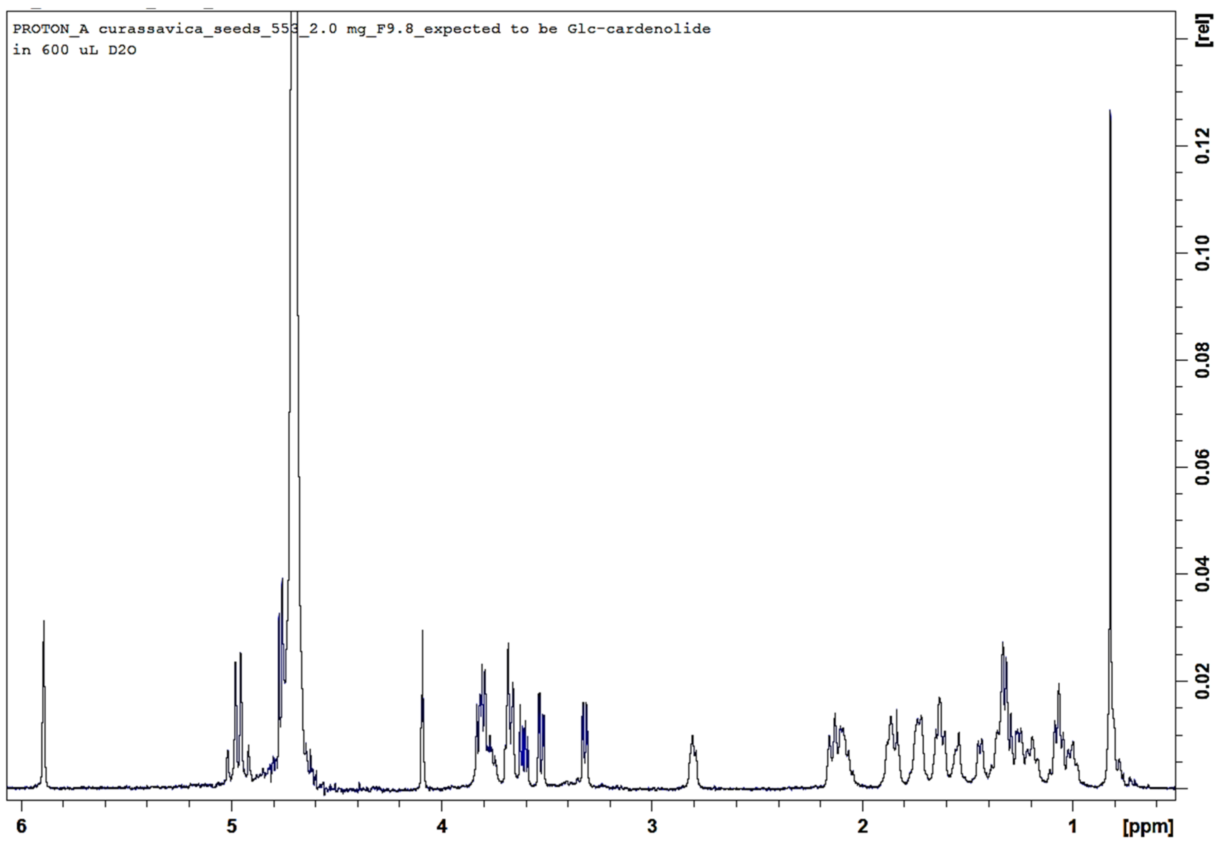

Figure S7. DEPTQ spectrum (125 MHz, D_2O) of compound **1**.

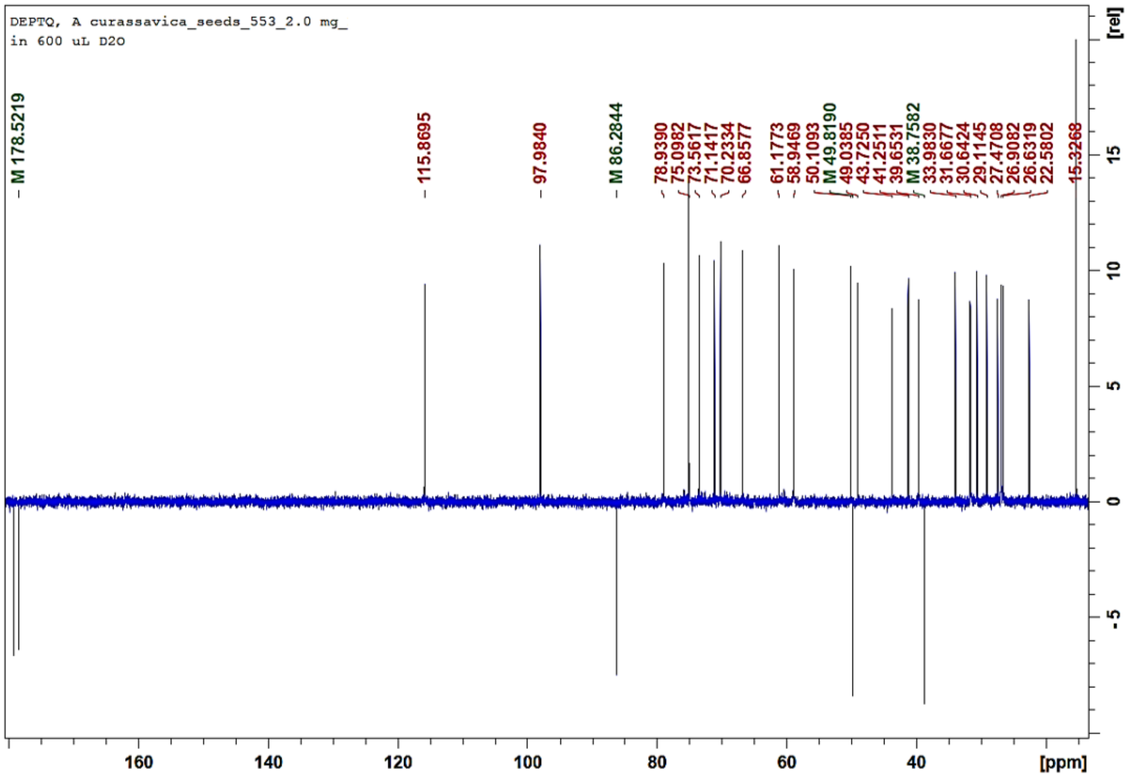

Figure S8. HSQC spectrum (500 MHz, D_2O) of compound **1.**

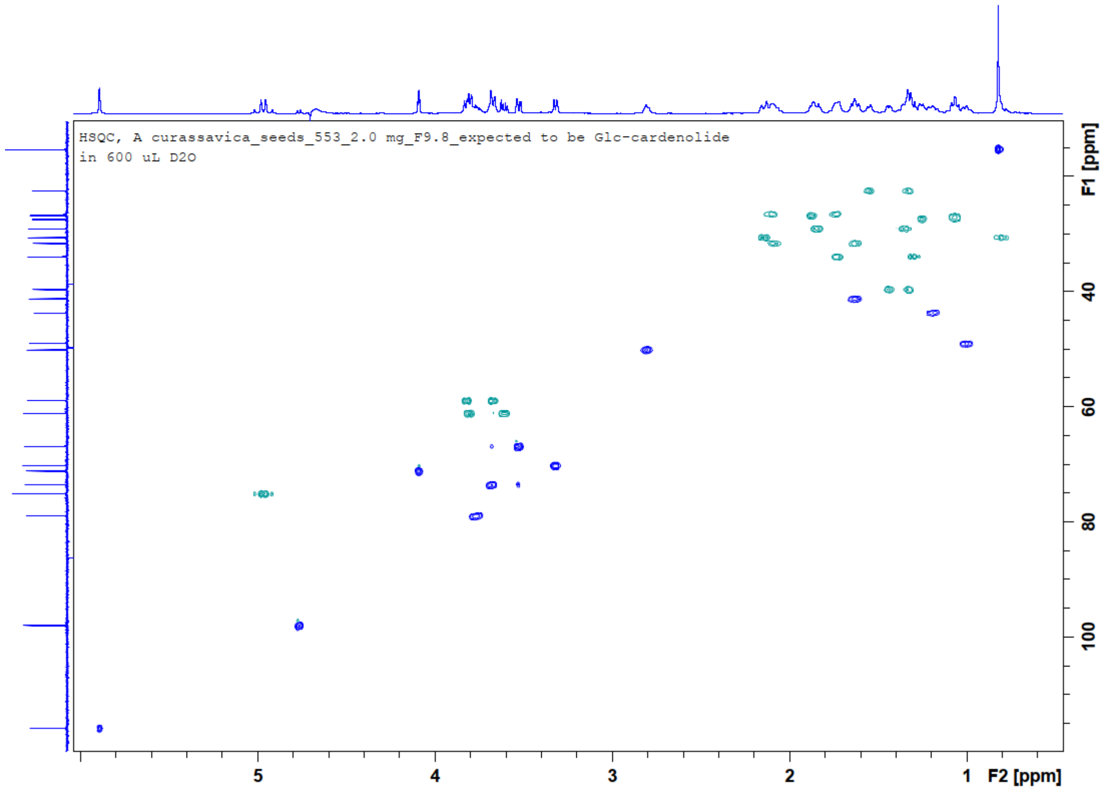

Figure S9. ^{1}H-^{1}H COSY spectrum (500 MHz, D_2O) of compound **1.**

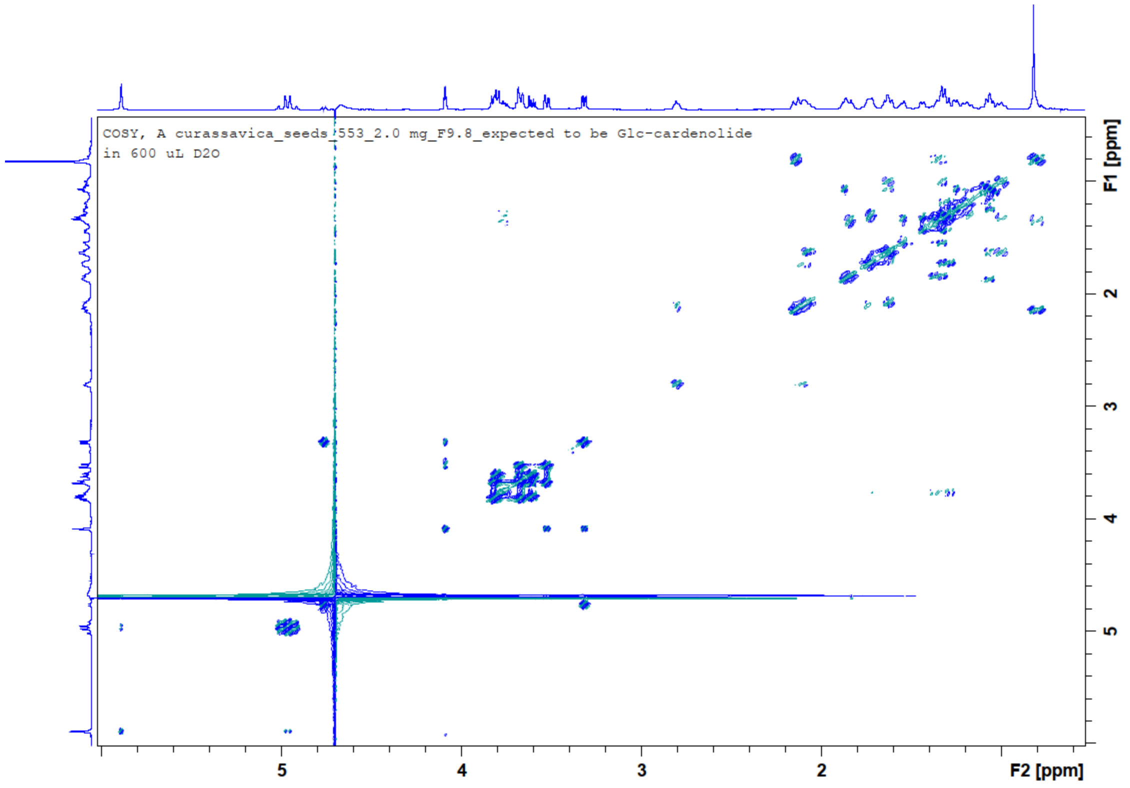

Figure S10. ^{1}H-^{13}C HMBC spectrum (500 MHz, D_2O) of compound **1.**

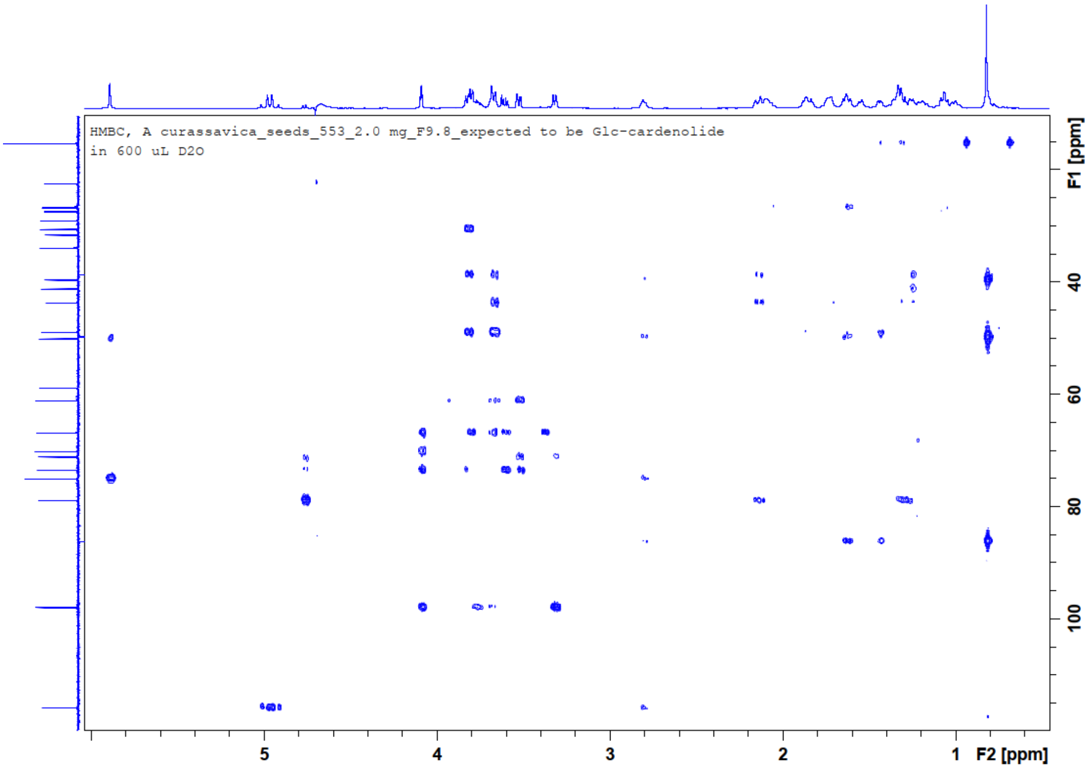

Figure S11. ROESY spectrum (500 MHz, D_2O) of compound **1.**

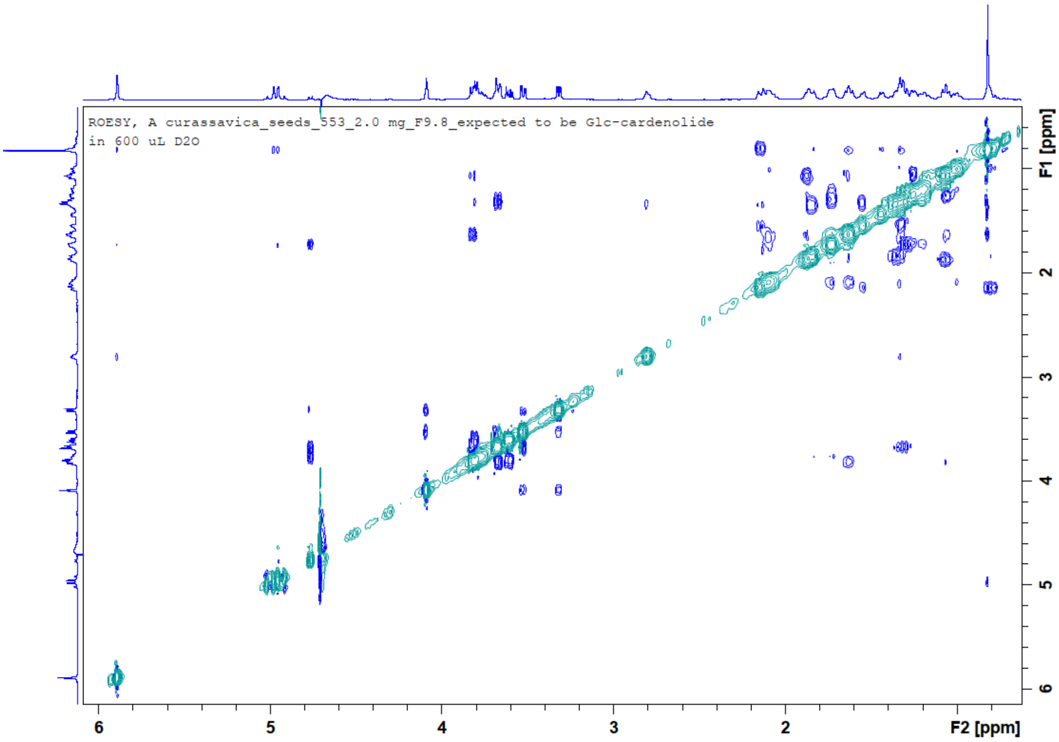

Figure S12. HSQC-TOCSY spectrum (500 MHz, D_2O) of compound **1.**

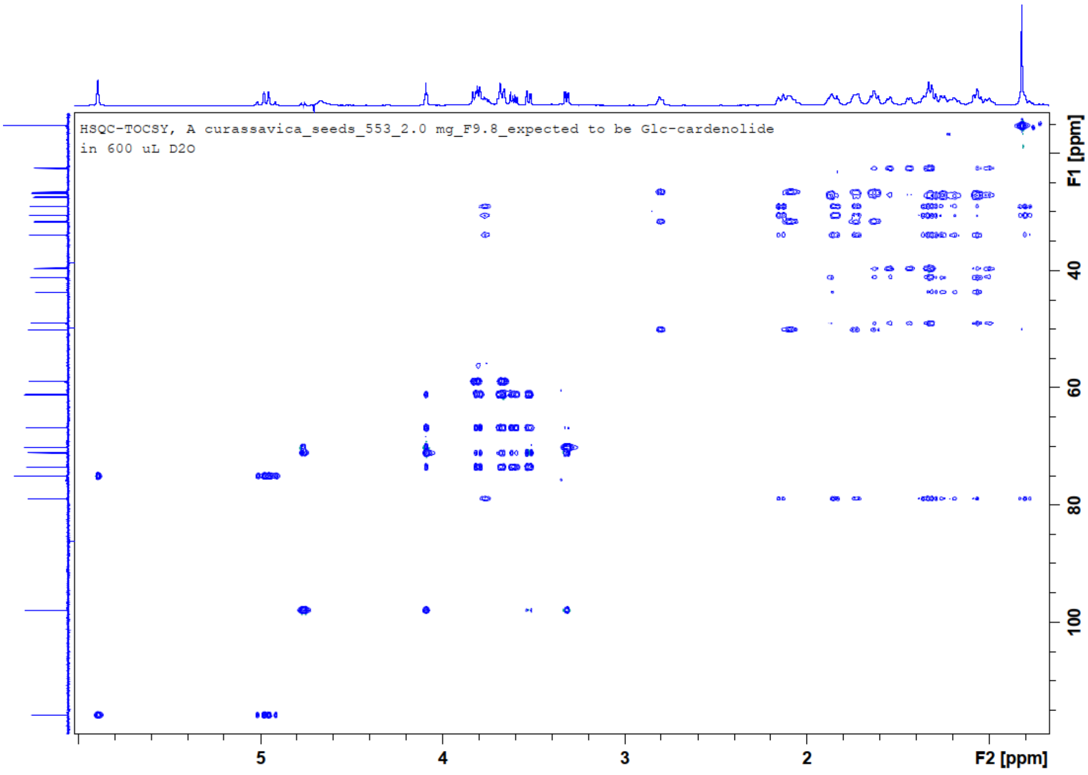

Figure S13. SELTOCSY spectrum (500 MHz, transmitter frequency at 3.609 ppm (H-6´), D_2O) of compound **1.**

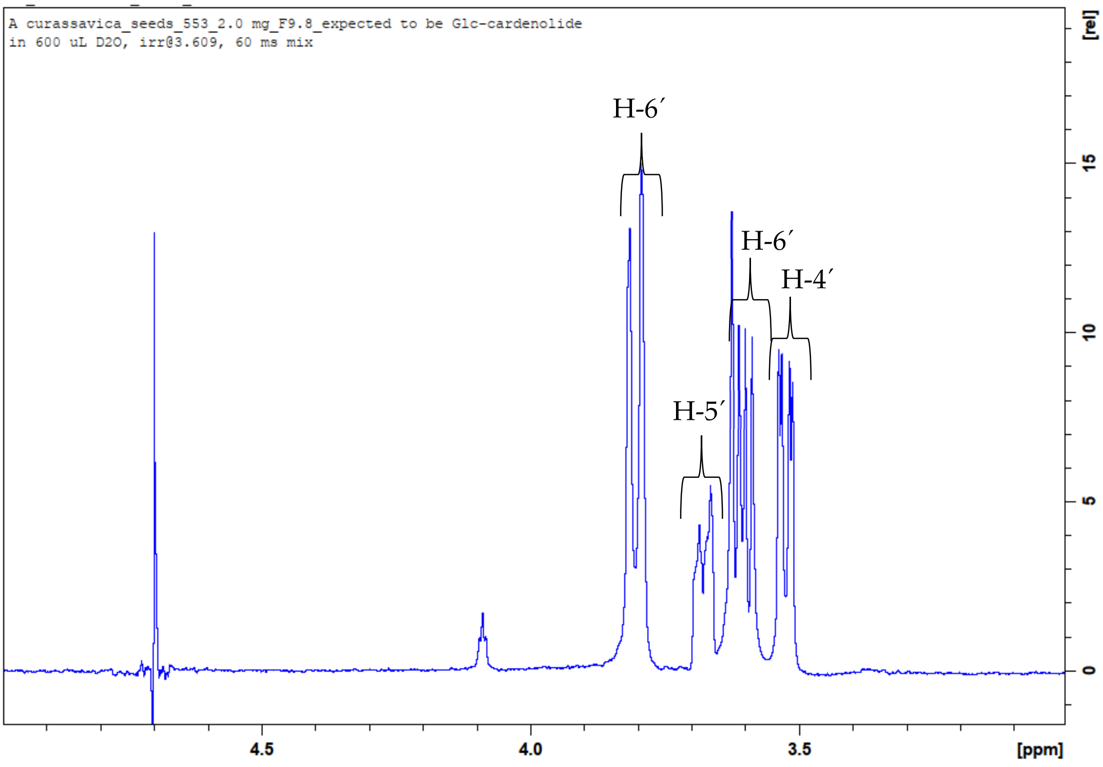

Figure S14. SELTOCSY spectrum (500 MHz, transmitter frequency at 3.765 ppm (H-3), D_2O) of compound **1.**

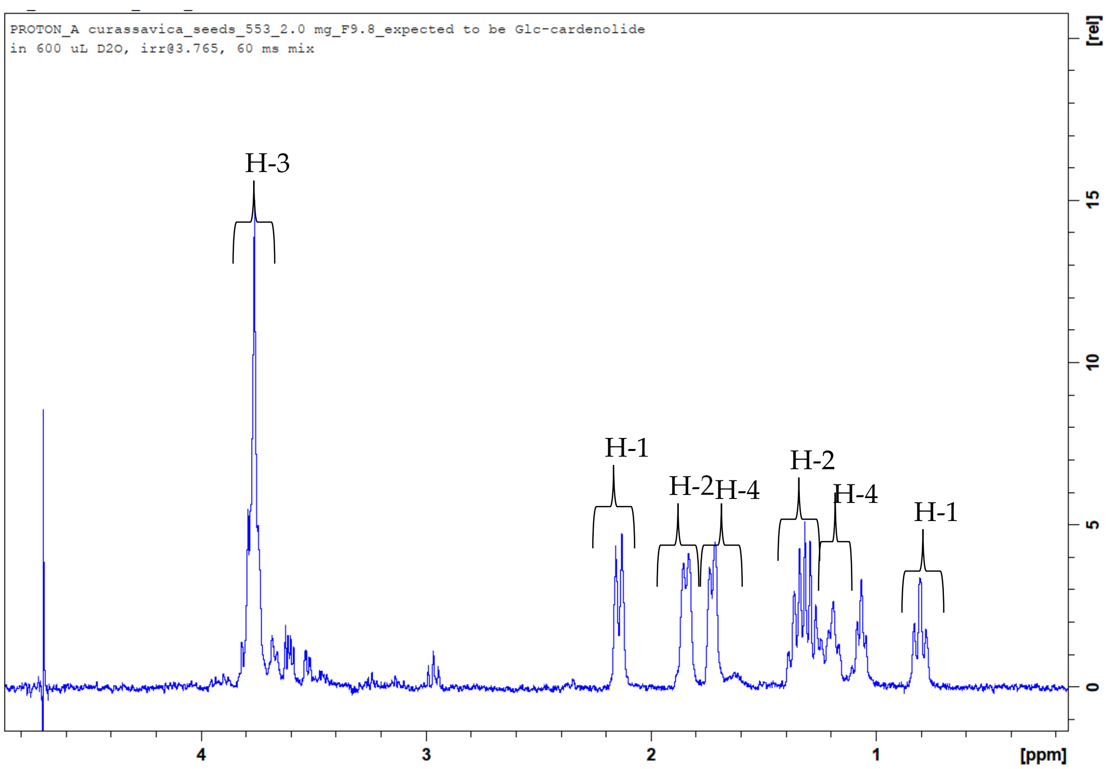

Figure S15: HR-ESI-MS spectrum of compound **2.**

Compound Spectrum List Report

Analysis Info

Analysis Name	\\filer\bird-lab\Users\Paola R-B\LCMS data\Asclepias extracts\715\alloglucoroglau 17.3.22 dilu 2_P1-D-4_01_239.d	Acquisition Date	17/03/2022 23:50:28
Method	paola_lcms_pos_checkuplc040221.m	Operator	MPI Jena
Sample Name	alloglucoroglau 17.3.22 dilu 2	Instrument	compact 8255754.20124
Comment			

Acquisition Parameter

Source Type	ESI	Ion Polarity	Positive	Set Nebulizer	1.8 Bar
Focus	Not active	Set Capillary	4500 V	Set Dry Heater	220 °C
Scan Begin	50 m/z	Set End Plate Offset	-500 V	Set Dry Gas	9.0 l/min
Scan End	1300 m/z	Set Charging Voltage	2000 V	Set Divert Valve	Waste
		Set Corona	0 nA	Set APCI Heater	0 °C

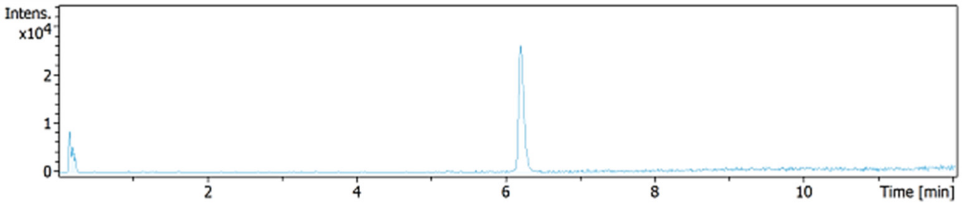

#	RT [min]	Area	Int. Type	I	S/N	Trace	Max. m/z	FWHM [min]
5	6.2	0		60745		EIC 715.3553 +All MS	282.2795	

Cmpd 5, 6.2 min

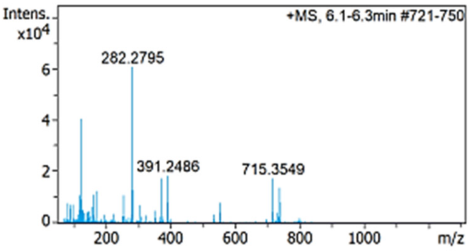

#	m/z	Res.	S/N	I	I %	FWHM
1	123.0556	9465	576363.1	40302	66.3	0.0130
2	163.1235	9869	159008.9	11119	18.3	0.0165
3	173.0790	9853	178719.4	12497	20.6	0.0176
4	282.2795	10774	868709.9	60745	100.0	0.0262
5	283.2826	10561	177397.7	12405	20.4	0.0268
6	284.2947	10235	183544.4	12834	21.1	0.0278
7	373.2380	10655	249696.2	17460	28.7	0.0350
8	391.2486	10880	264242.6	18477	30.4	0.0360
9	715.3549	11565	250492.6	17516	28.8	0.0619
10	737.3366	11616	196734.8	13757	22.6	0.0635

Figure S16. UV spectrum of compound **2.**

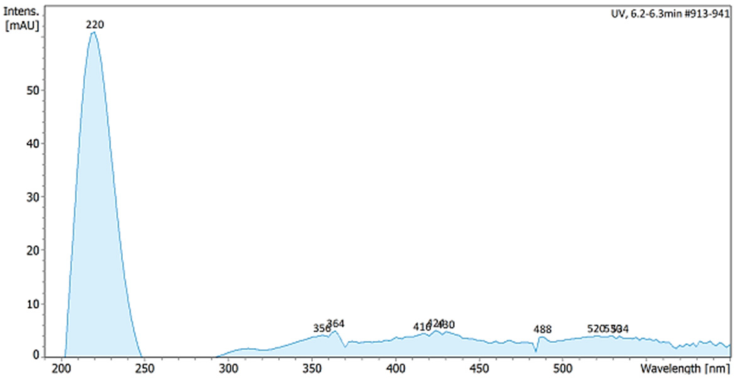

Figure S17: Compound **2**-NMR shift assignment

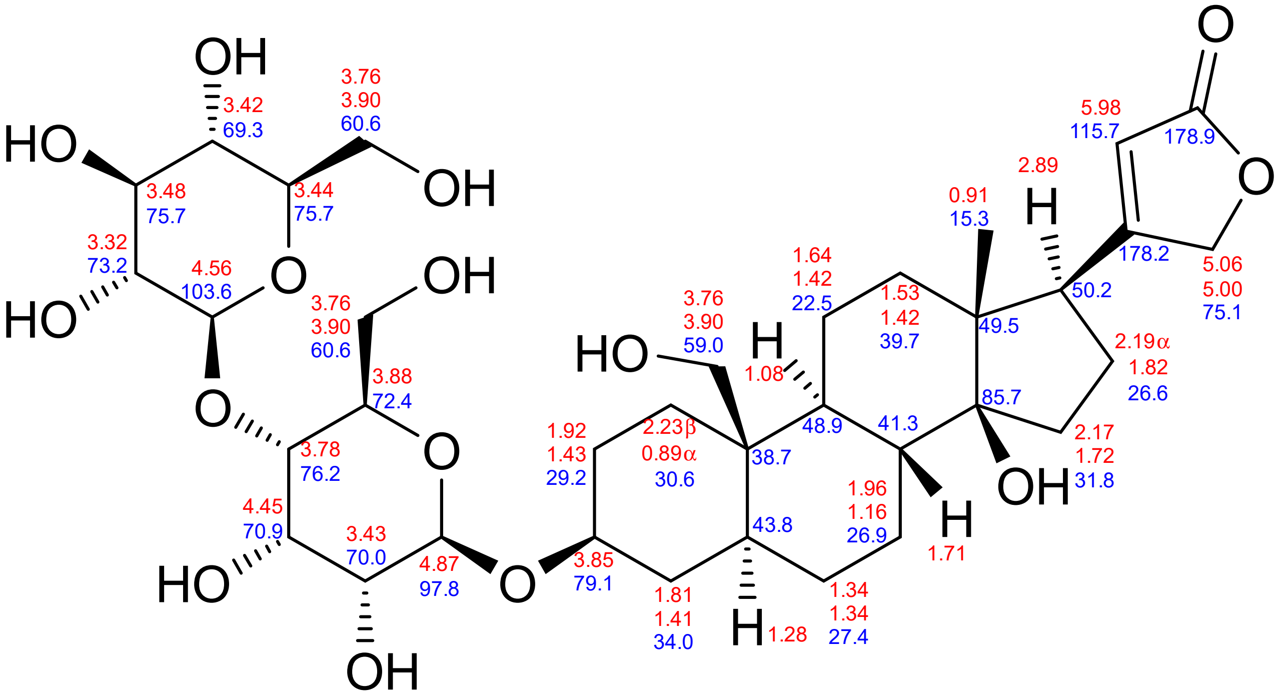

3β-[(4-O-β-D-glucopyranosyl-β-D-allopyranosyl)oxy]-14,19-dihydroxy-5α-card-20(22)-enolide

Figure S18. ^{1}H-NMR spectrum (500 MHz, D_2O) of compound **2**.

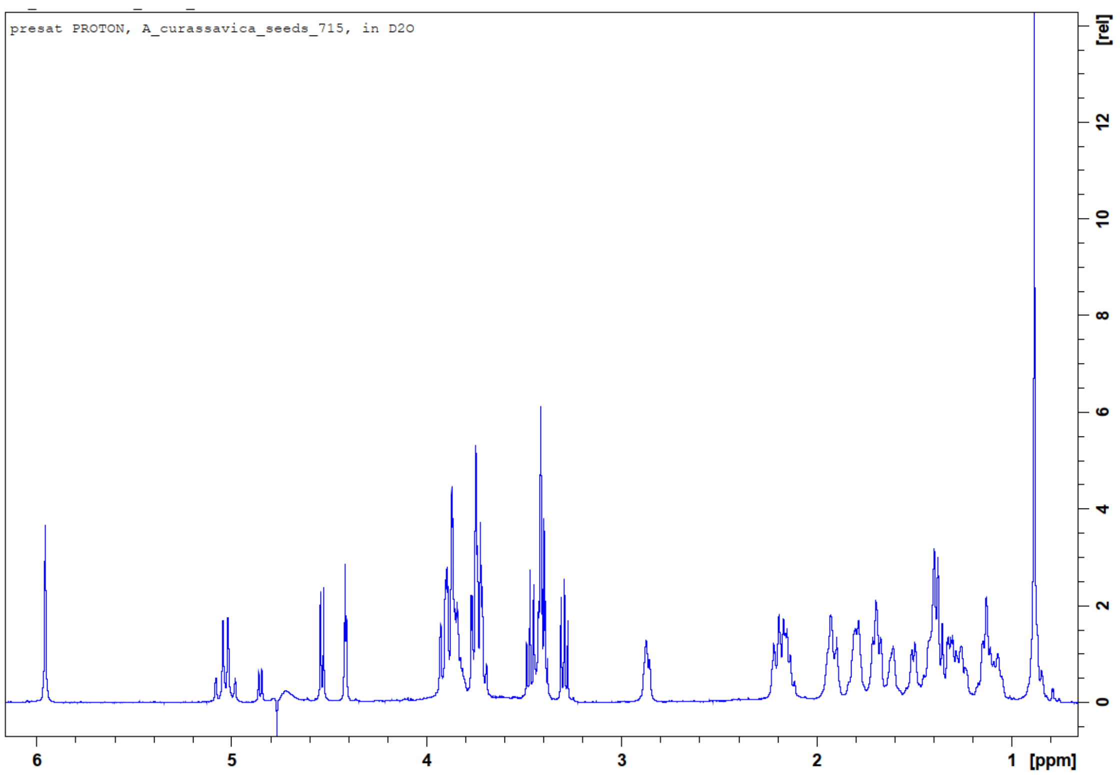

Figure S19. DEPTQ spectrum (125 MHz, D2O) of compound **2**.

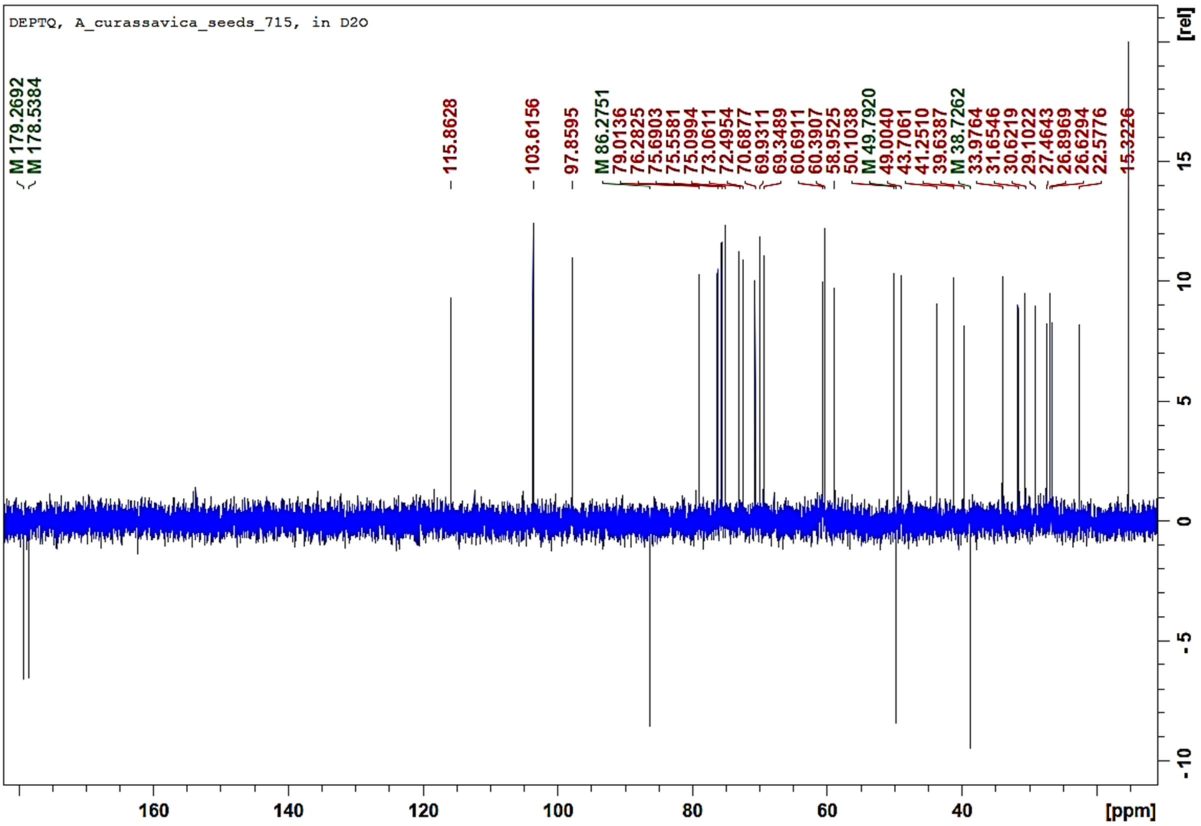

Figure S20. HSQC spectrum (500 MHz, D_2O) of compound **2.**

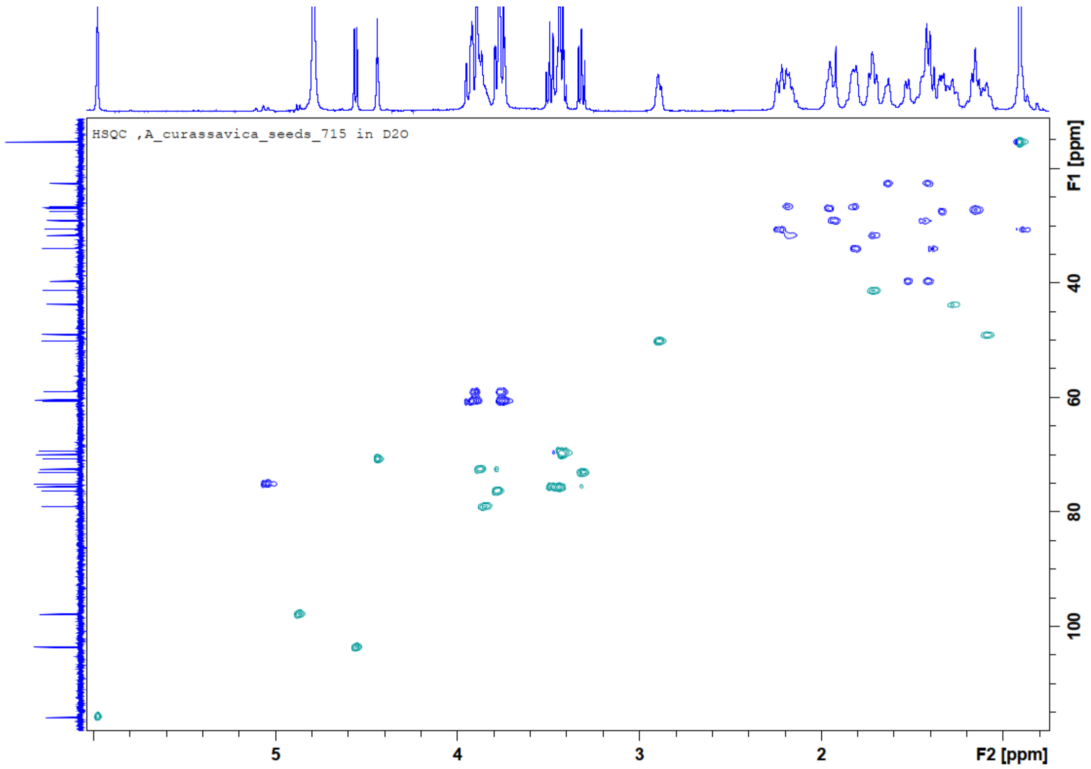

Figure S21. ^1H-^1H COSY spectrum (500 MHz, D_2O) of compound **2.**

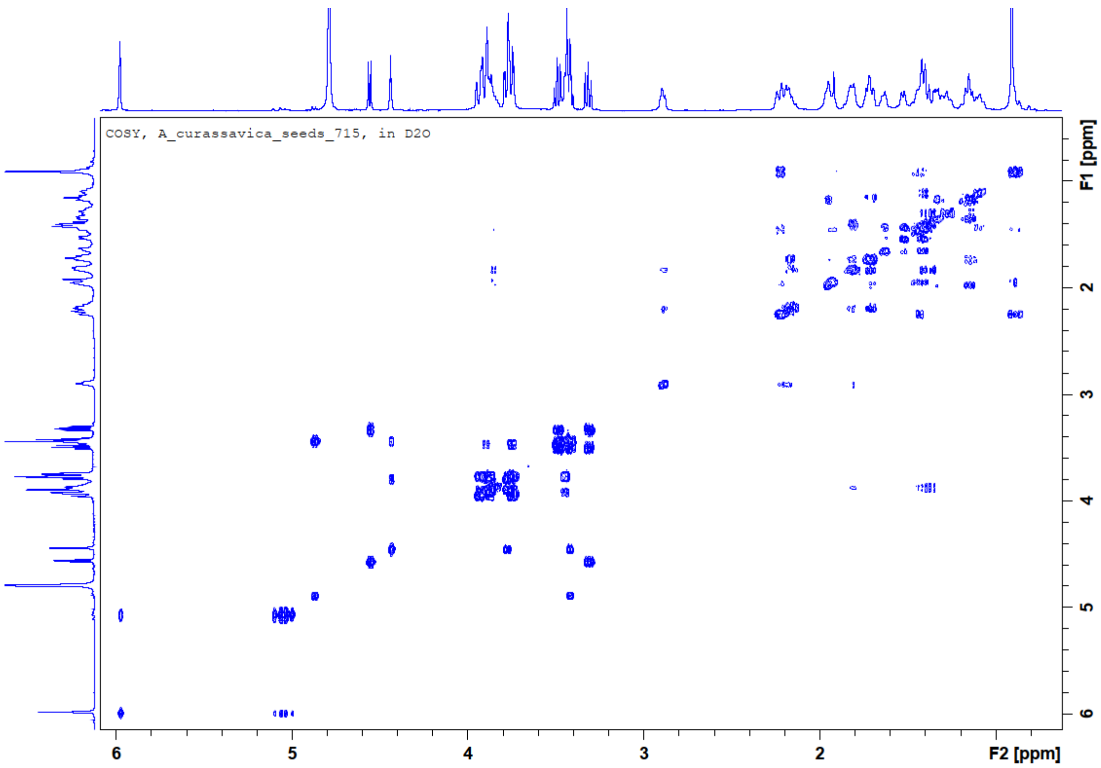

Figure S22. ^{1}H-^{13}C HMBC spectrum (500 MHz, D_2O) of compound **2.**

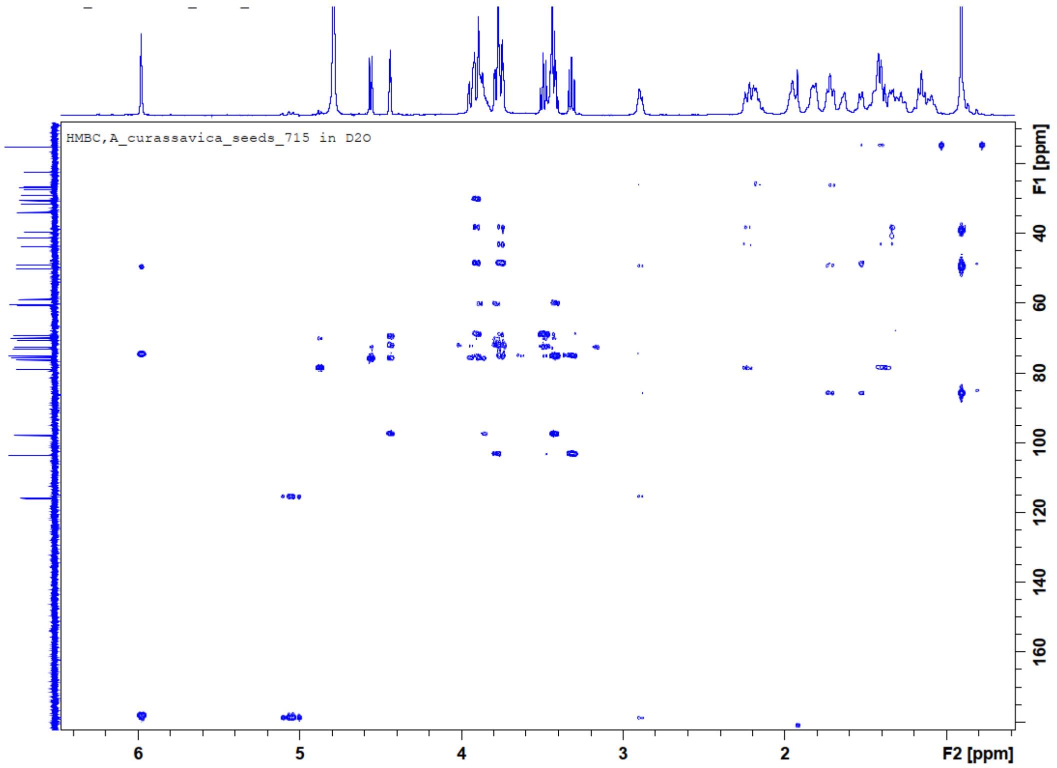

Figure S23. ROESY spectrum (500 MHz, D_2O) of compound **2.**

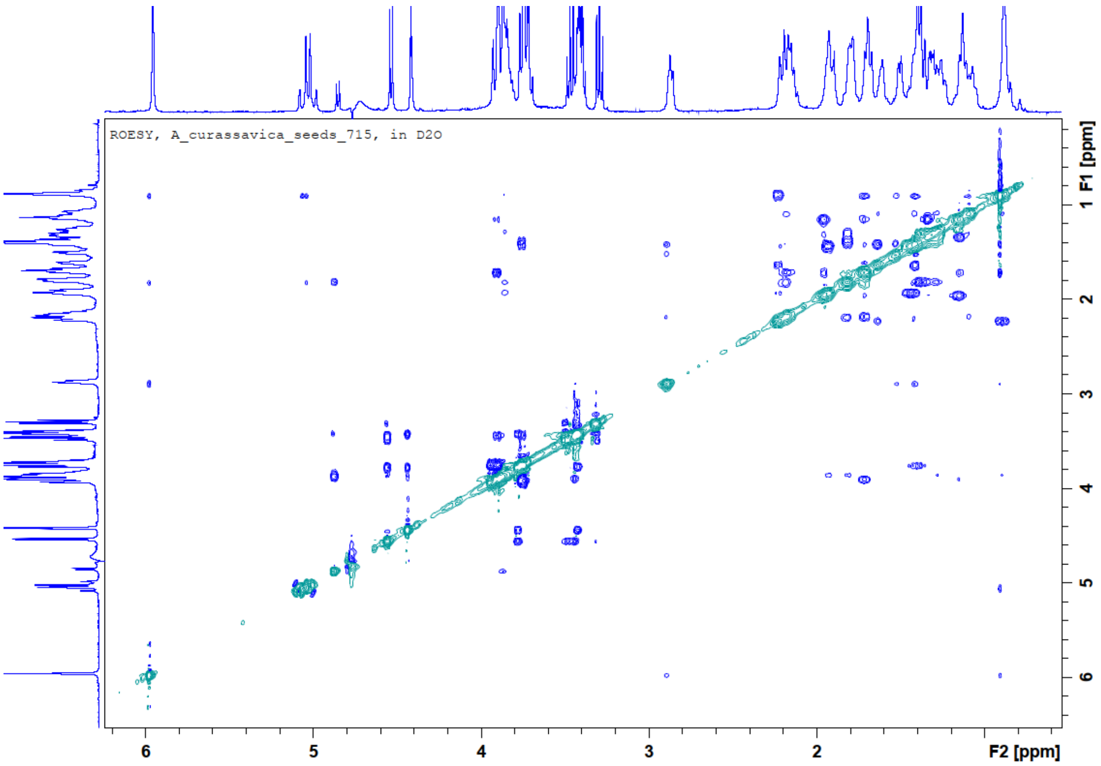

Figure S24. HSQC-TOCSY spectrum (500 MHz, D_2O) of compound **2.**

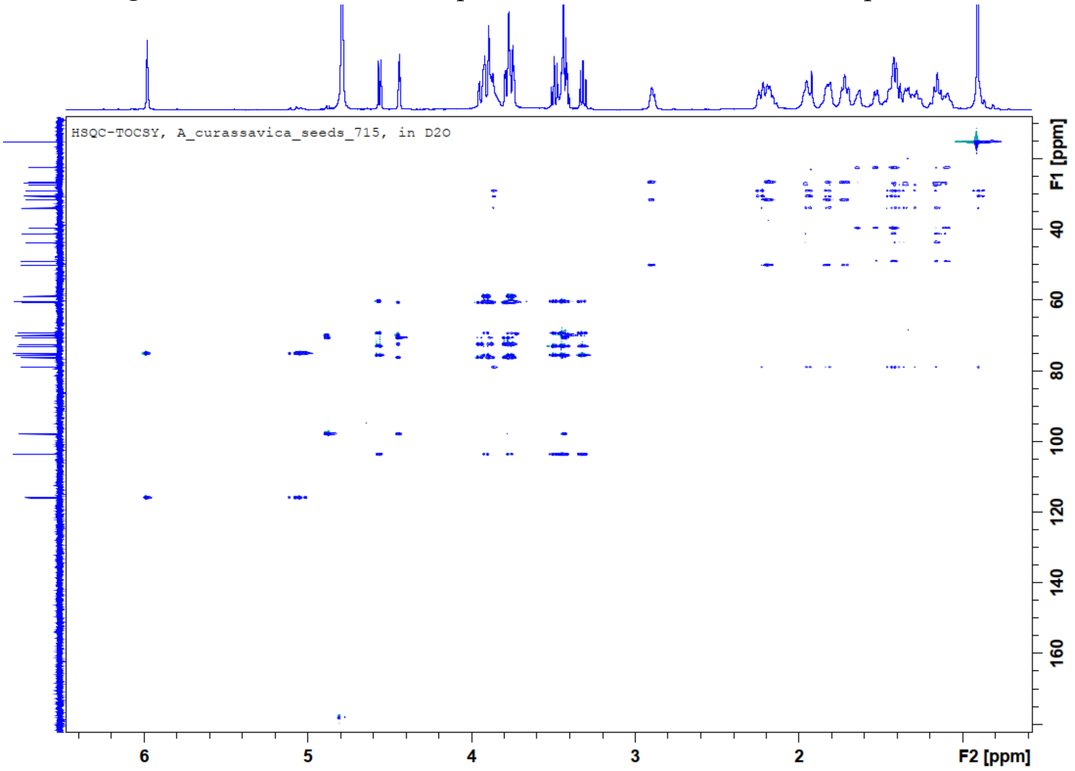

Figure S25. SELTOCSY spectrum (500 MHz, transmitter frequency at 4.87 ppm (H-1´), D_2O) of compound **2.**

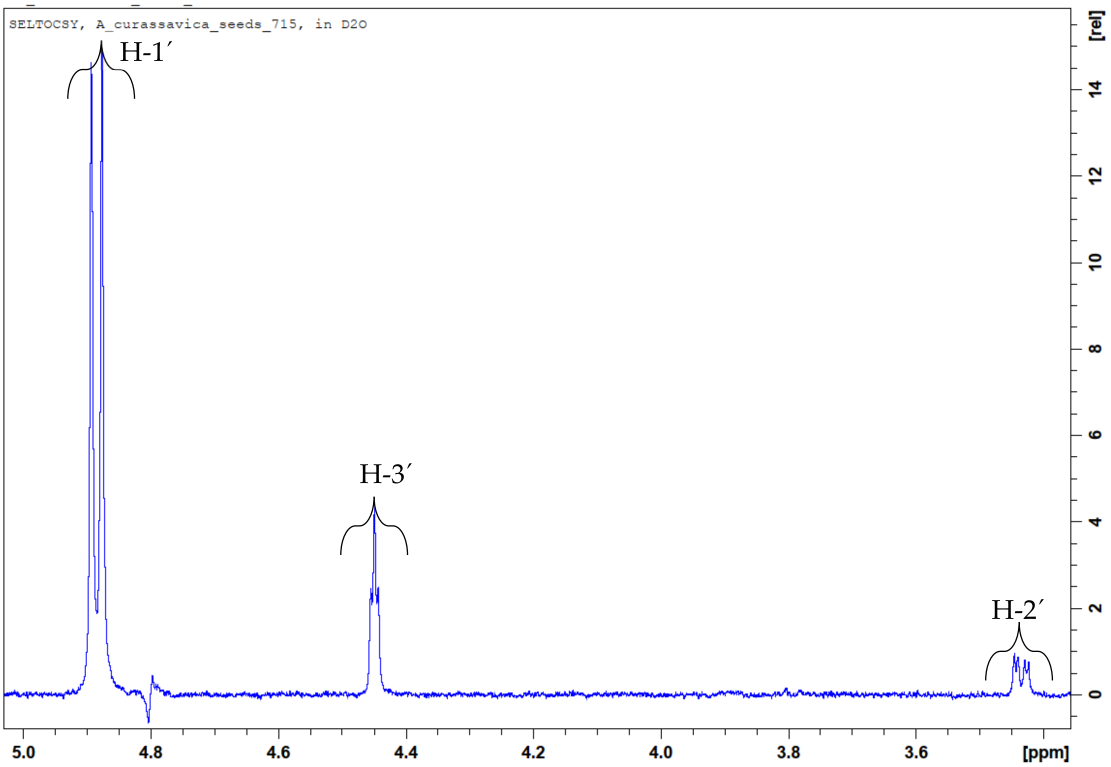

Figure S26. SELTOCSY spectrum (500 MHz, transmitter frequency at 4.45 ppm (H-1´´), D_2O) of compound **2.**

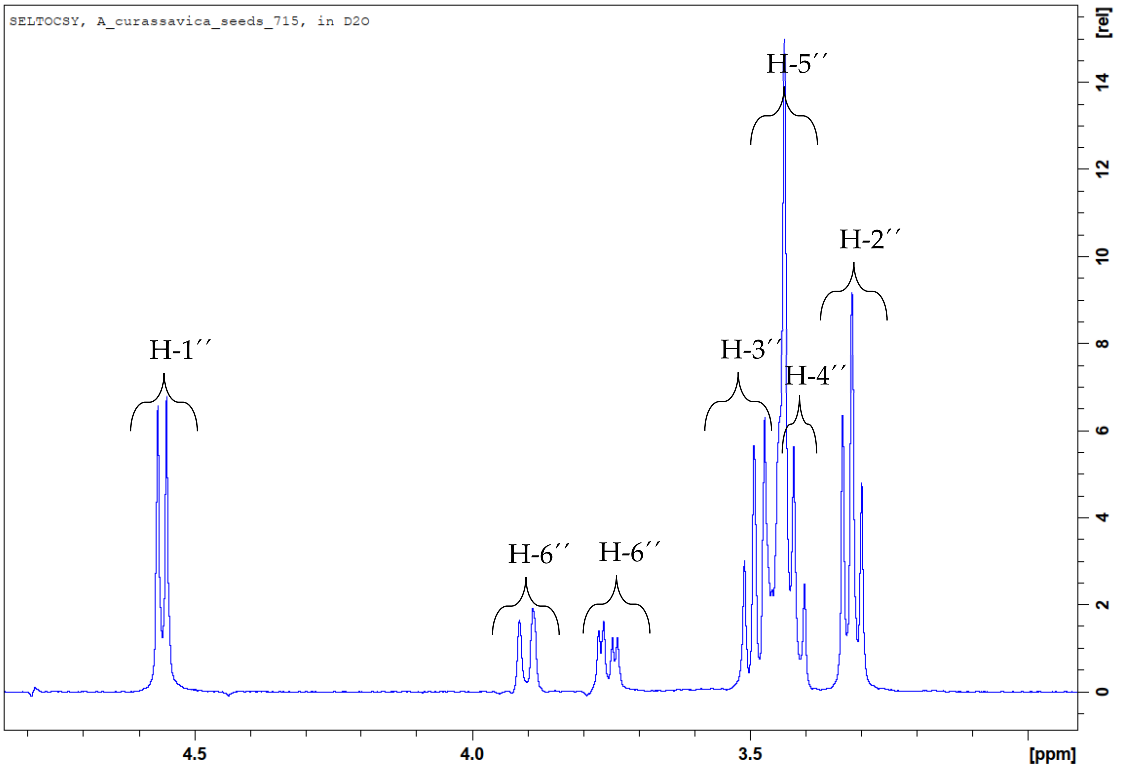

Figure S27: HR-ESI-MS spectrum of compound **3.**

Compound Spectrum List Report

Analysis Info

Analysis Name	\\filer\bird-lab\Users\Paola R-B\LCMS data\Asclepias extracts\693\OHglucocalotrop 17.3.22 dilu 2_P1-E-5_01_250.d	Acquisition Date	18/03/2022 03:06:11
Method	paola_lcms_pos_checkuplc040221.m	Operator	MPI Jena
Sample Name	OHglucocalotrop 17.3.22 dilu 2	Instrument	compact 8255754.20124
Comment			

Acquisition Parameter

Source Type	ESI	Ion Polarity	Positive	Set Nebulizer	1.8 Bar
Focus	Not active	Set Capillary	4500 V	Set Dry Heater	220 °C
Scan Begin	50 m/z	Set End Plate Offset	-500 V	Set Dry Gas	9.0 l/min
Scan End	1300 m/z	Set Charging Voltage	2000 V	Set Divert Valve	Waste
		Set Corona	0 nA	Set APCI Heater	0 °C

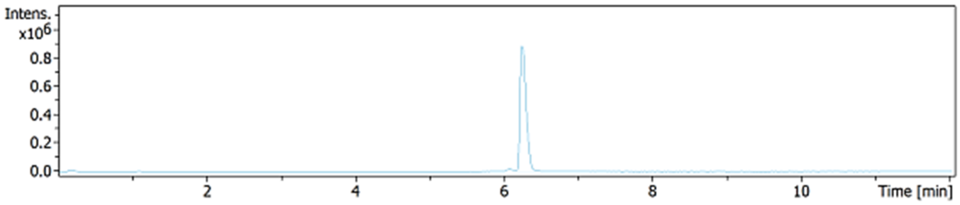

#	RT [min]	Area	Int. Type	I	S/N	Trace	Max. m/z	FWHM [min]
1	6.2	0		870479		EIC 693.3114 +All MS	693.3113	

Cmpd 1, 6.2 min

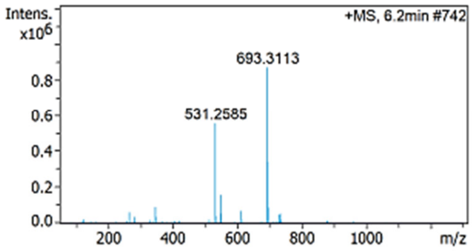

#	m/z	Res.	S/N	I	I %	FWHM
1	266.1326	11730	491.6	60963	7.0	0.0227
2	347.1585	12481	741.5	91945	10.6	0.0278
3	531.2585	11453	4492.6	557083	64.0	0.0464
4	532.2620	11322	1450.9	179915	20.7	0.0470
5	549.2693	11554	1278.0	158468	18.2	0.0475
6	612.2844	12184	550.4	68246	7.8	0.0503
7	693.3113	11752	7020.0	870479	100.0	0.0590
8	694.3145	11731	2766.1	342992	39.4	0.0592
9	695.3174	11857	728.1	90286	10.4	0.0586
10	733.3048	11744	426.0	52824	6.1	0.0624

Figure S28: UV spectrum of compound **3.**

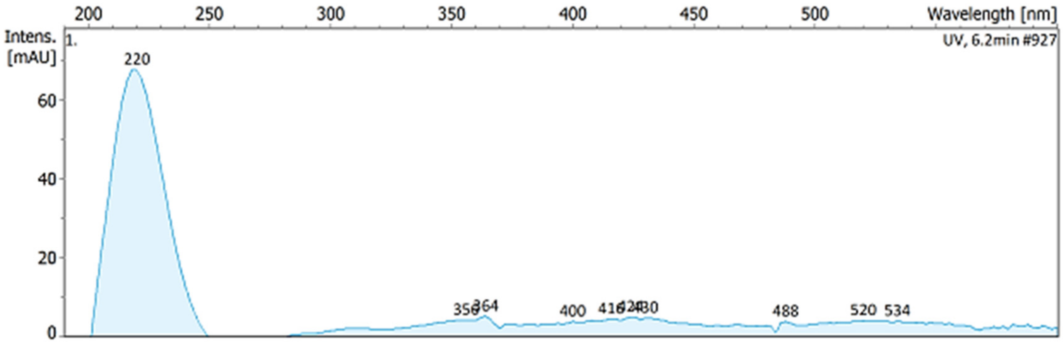

Figure S29: Compound **3**-NMR shift assignment

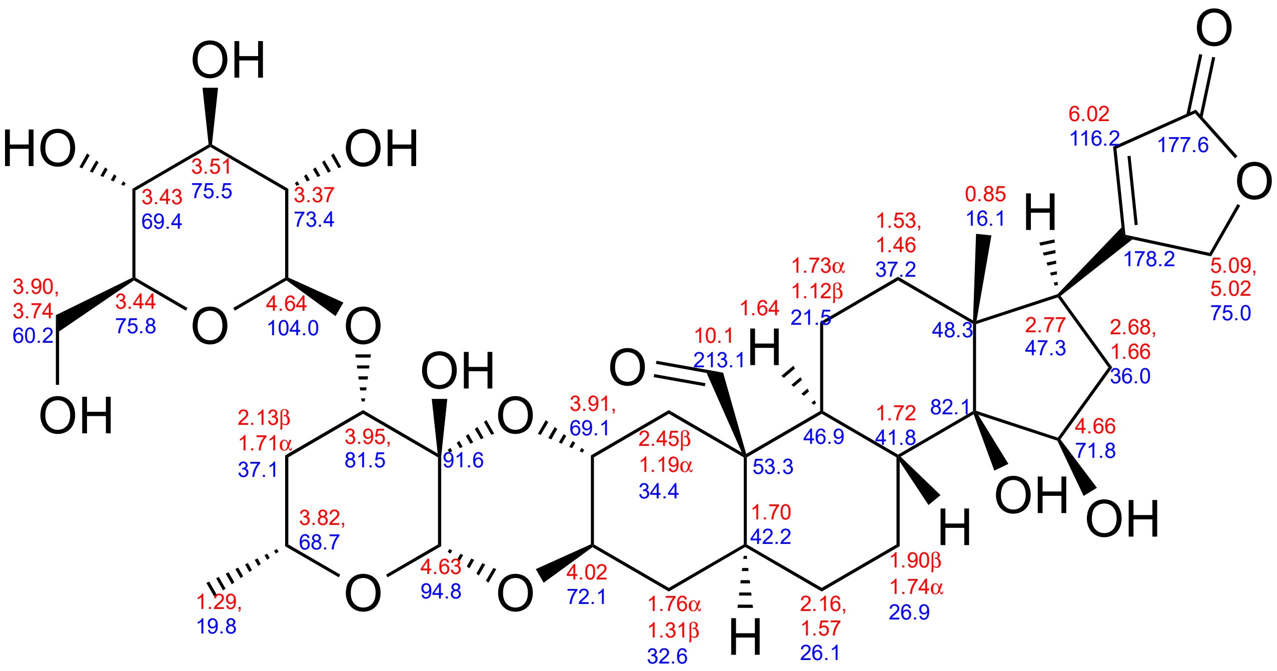

(1*R*,3*R*,3a*S*,3b*R*,6a*R*,7a*S*,9*R*,11*S*,11a*S*,12a*R*,13a*R*,13b*S*,15a*R*)-13a-formyl-3,3a,11a-trihydroxy-9,15a-dimethyl-1-(5-oxo-2,5-dihydrofuran-3-yl)icosahydro-1*H*,7a*H*-cyclopenta[7,8]phenanthro[2,3*b*]pyrano[3,2-*e*][1,4]dioxin-11-yl β-L-glucopyranoside

Figure S30. ^{1}H-NMR spectrum (500 MHz, D_2O) of compound **3**.

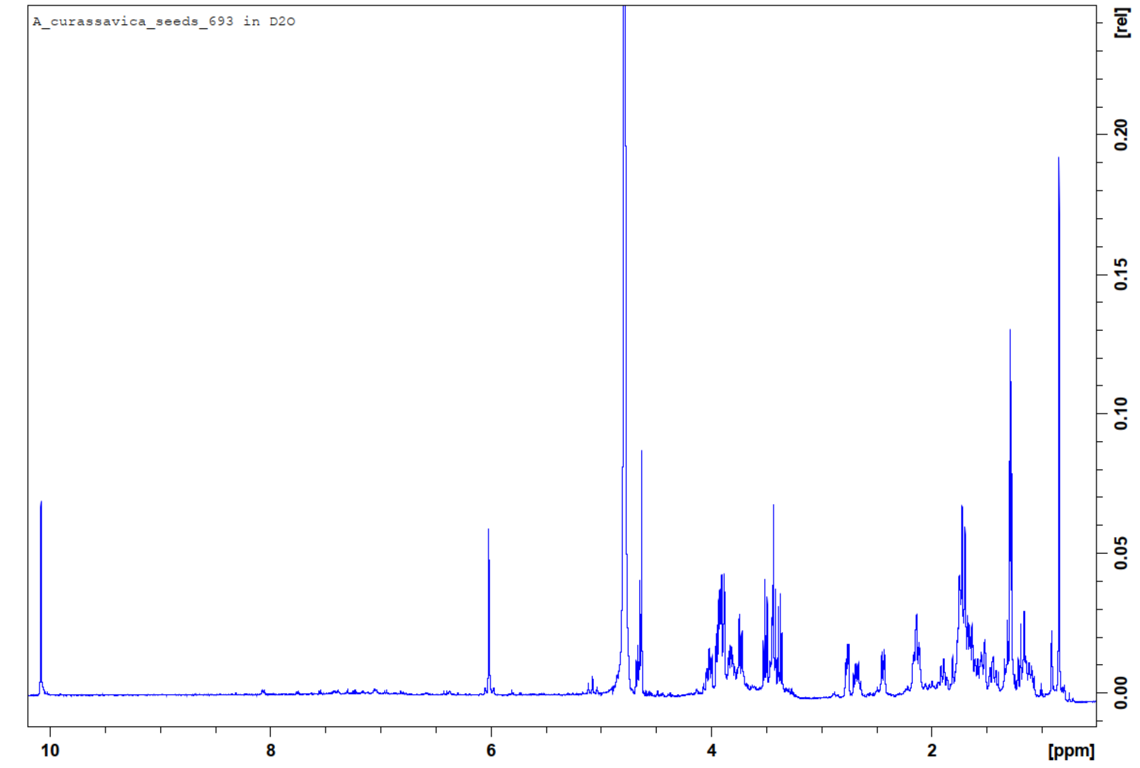

Figure S31. DEPTQ spectrum (125 MHz, D_2O) of compound **3**.

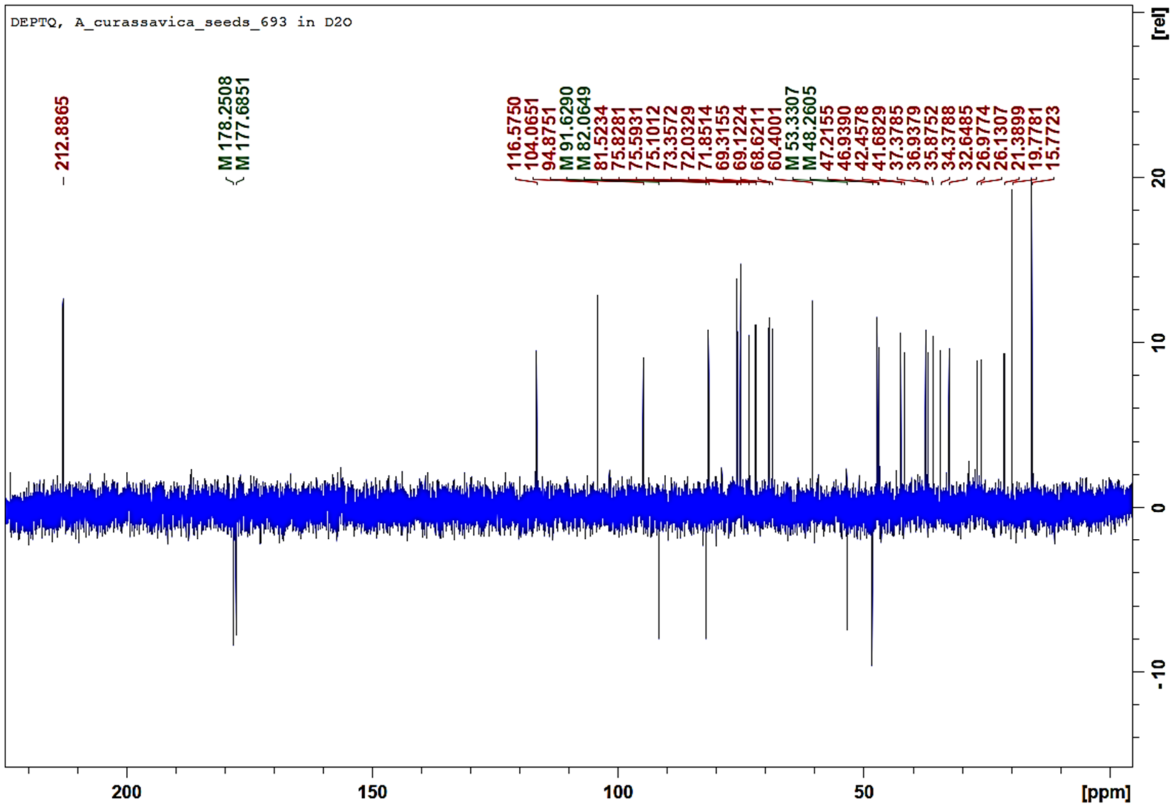

Figure S32. HSQC spectrum (500 MHz, D_2O) of compound **3.**

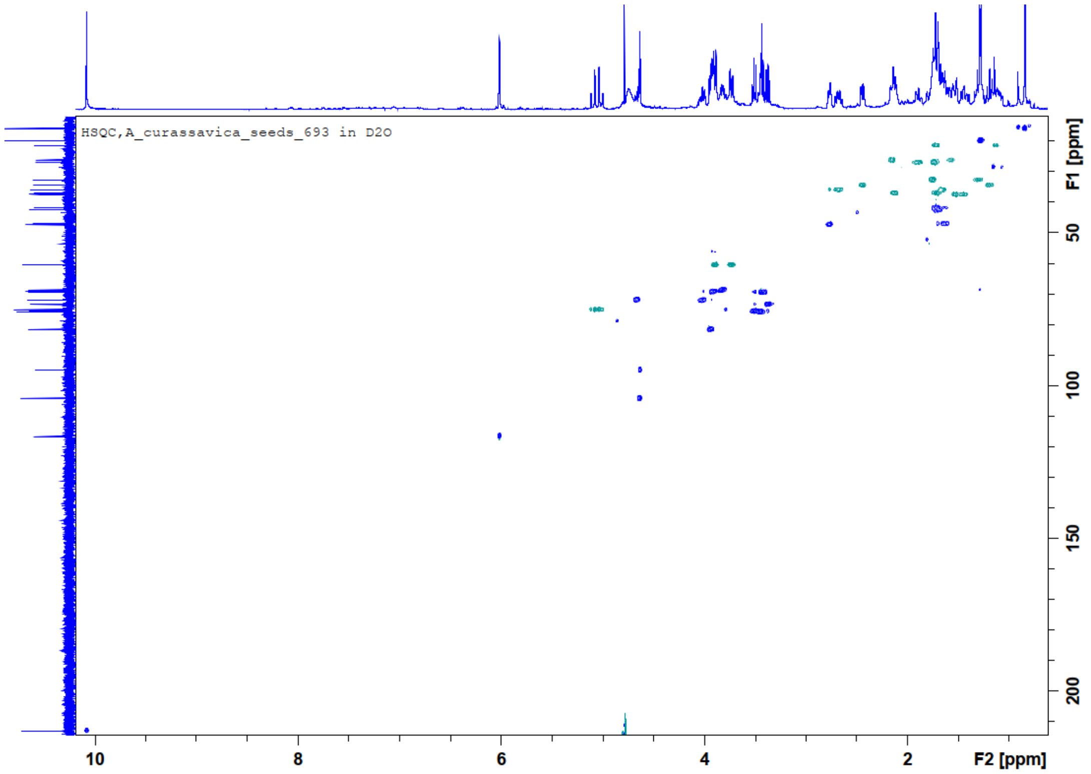

Figure S33. ^1H-^1H COSY spectrum (500 MHz, D_2O) of compound **3.**

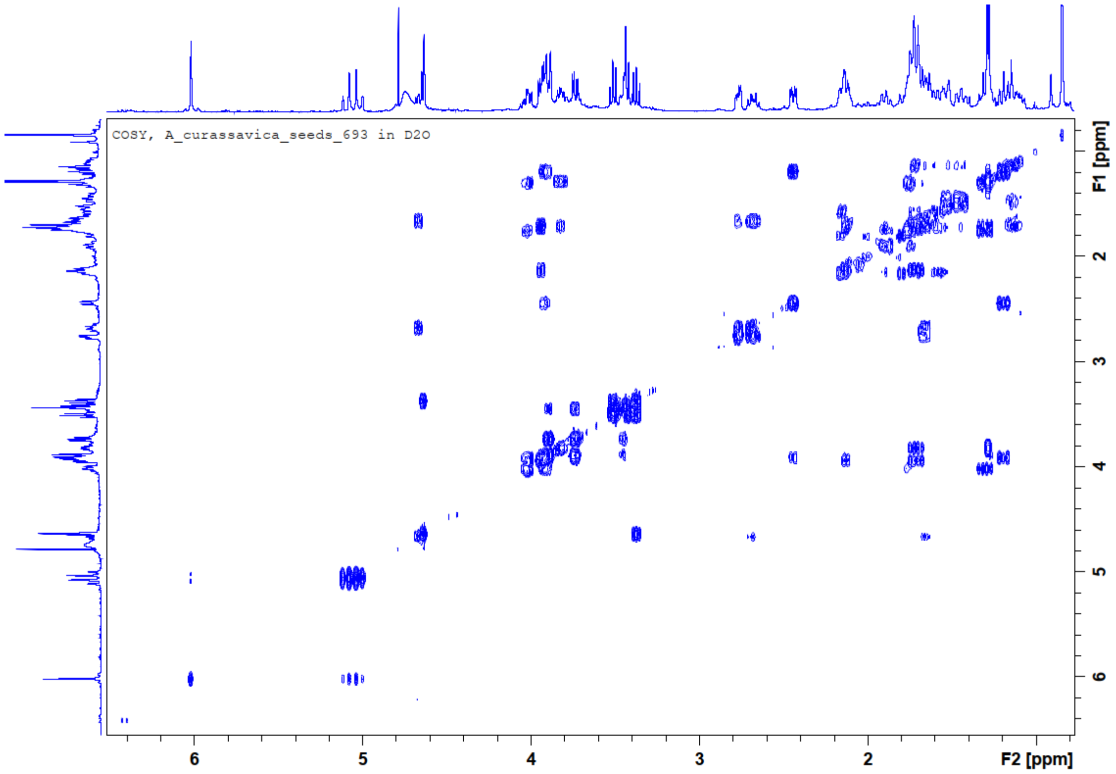

Figure S34. ^1H-^{13}C HMBC spectrum (500 MHz, D_2O) of compound **3.**

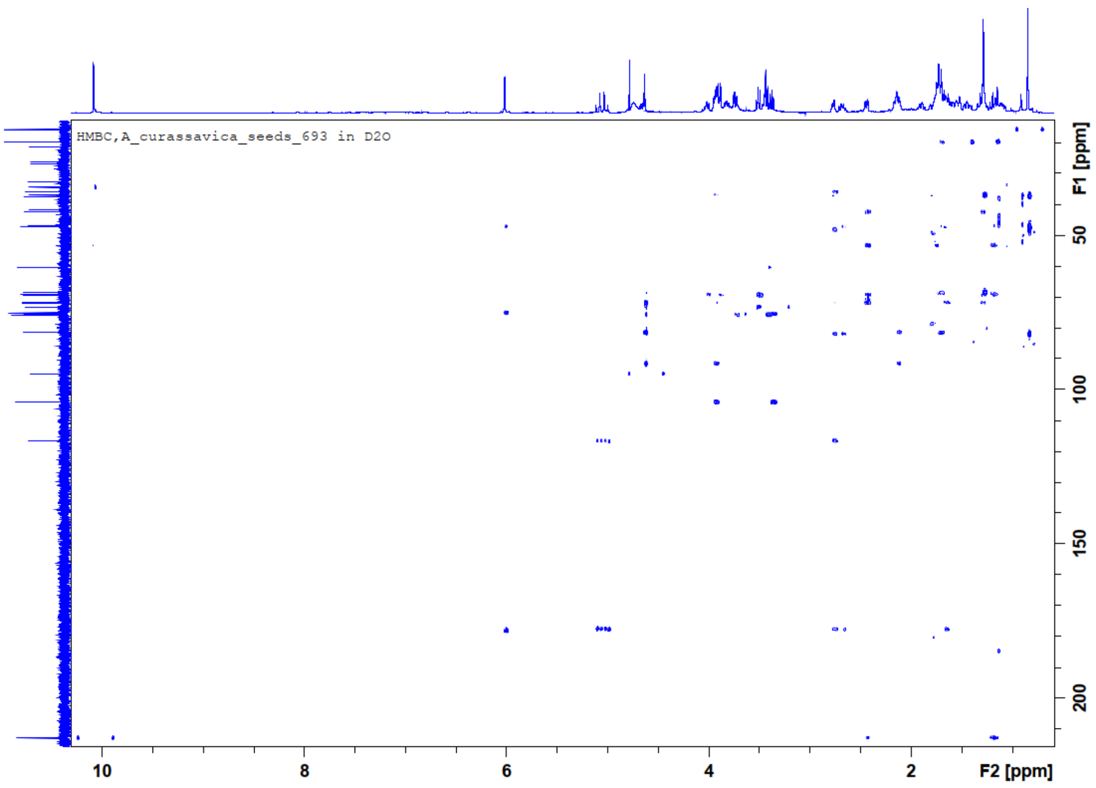

Figure S35. ROESY spectrum (500 MHz, D_2O) of compound **3.**

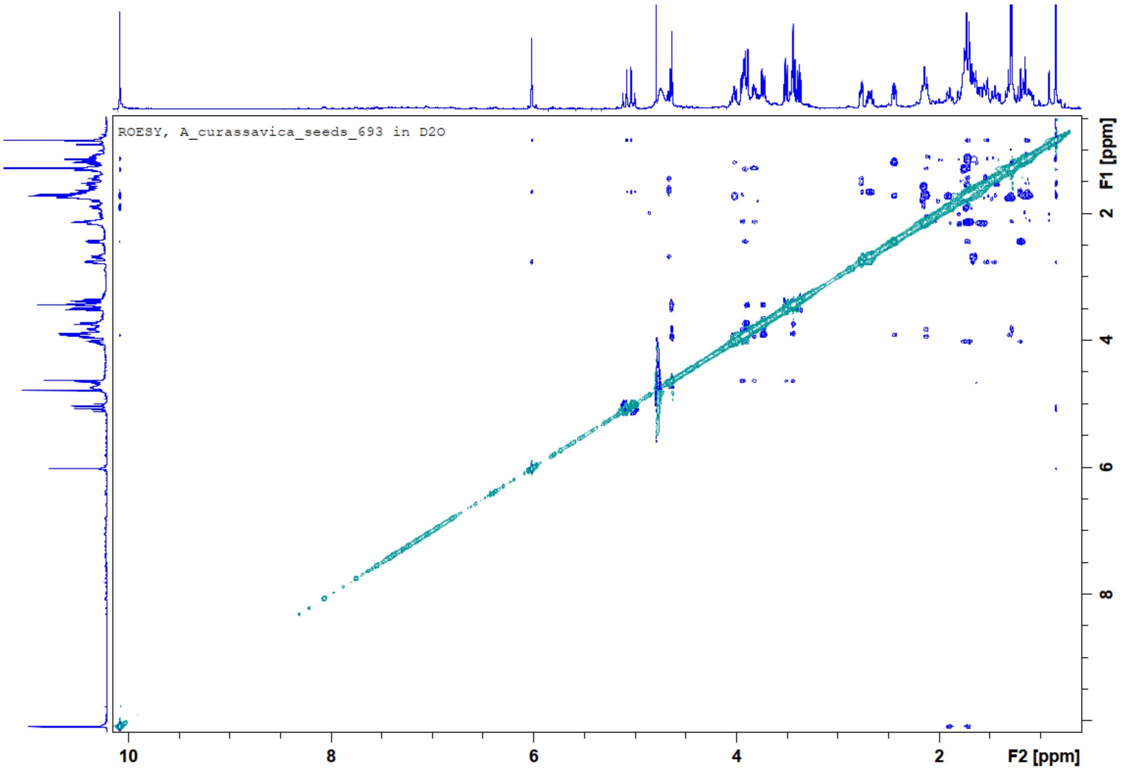

Figure S36. HSQC-TOCSY spectrum (500 MHz, D_2O) of compound **3.**

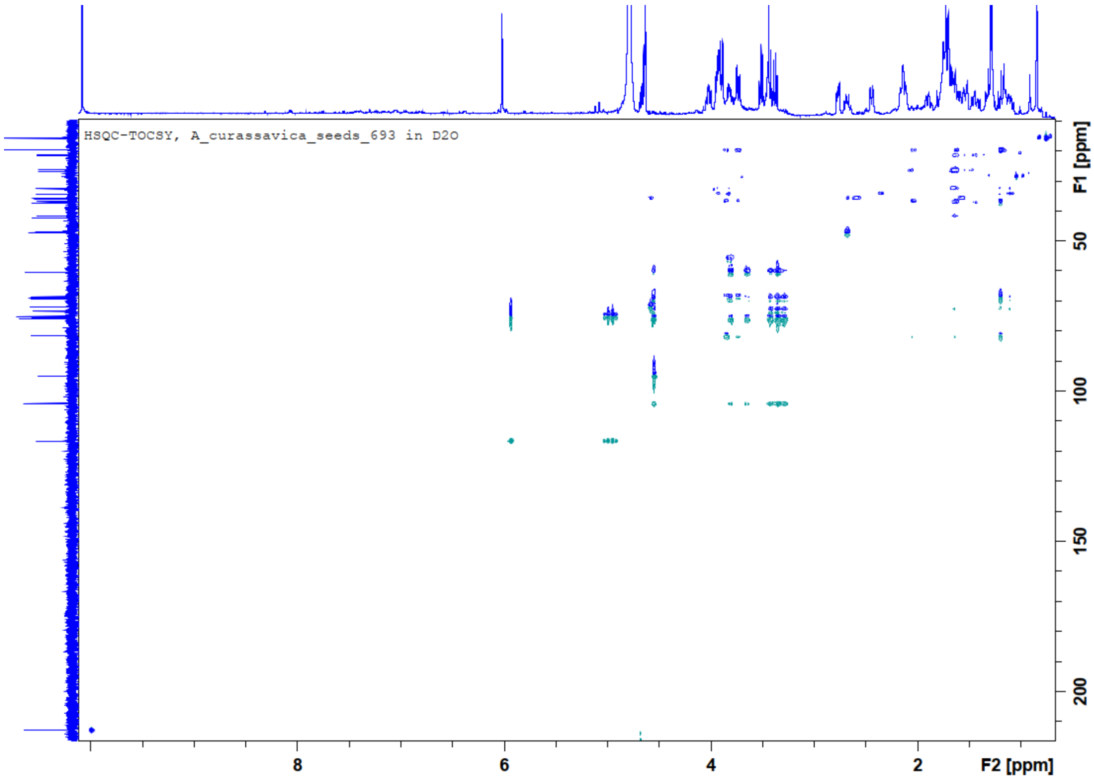

HSQC-TOCSY, A_curassavica_seeds_693 in D2O
F1 [ppm]
50
100
150
200
8
6
4
2
F2 [ppm]

Figure S37: HR-ESI-MS spectrum of compound **4.**

Compound Spectrum List Report

Analysis Info

Analysis Name	\\filer\bird-lab\Users\Paola R-B\LCMS data\Asclepias extracts\569 calibration\OHglucocoroglau 17.3.22 dilu 2_P1-C-3_01_228.d	Acquisition Date	17/03/2022 20:34:44
Method	paola_lcms_pos_checkuplc040221.m	Operator	MPI Jena
Sample Name	OHglucocoroglau 17.3.22 dilu 2	Instrument	compact 8255754.20124
Comment			

Acquisition Parameter

Source Type	ESI	Ion Polarity	Positive	Set Nebulizer	1.8 Bar
Focus	Not active	Set Capillary	4500 V	Set Dry Heater	220 °C
Scan Begin	50 m/z	Set End Plate Offset	-500 V	Set Dry Gas	9.0 l/min
Scan End	1300 m/z	Set Charging Voltage	2000 V	Set Divert Valve	Waste
		Set Corona	0 nA	Set APCI Heater	0 °C

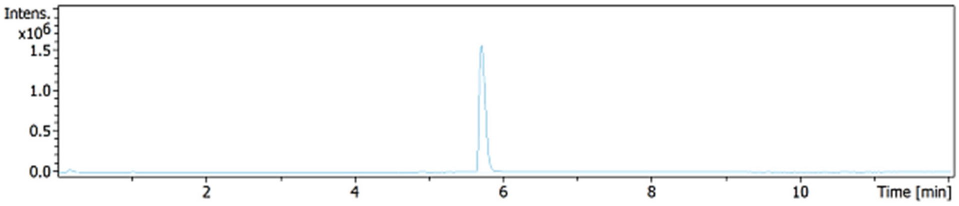

#	RT [min]	Area	Int. Type	I	S/N	Trace	Max. m/z	FWHM [min]
3	5.7	0		1523101		EIC 569.2970 +All MS	569.2970	

Cmpd 3, 5.7 min

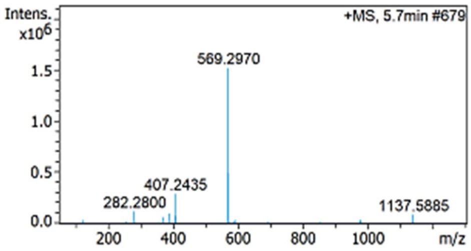

#	m/z	Res.	S/N	I	I %	FWHM
1	282.2800	10786	977.3	121180	8.0	0.0262
2	371.2224	10899	512.0	63484	4.2	0.0341
3	389.2331	10974	785.1	97351	6.4	0.0355
4	407.2435	11084	2380.8	295217	19.4	0.0367
5	408.2473	11000	603.3	74804	4.9	0.0371
6	569.2970	11398	12283.1	1523101	100.0	0.0499
7	570.3002	11553	4006.4	496797	32.6	0.0494
8	571.3036	11400	930.1	115328	7.6	0.0501
9	1137.5885	11889	703.1	87189	5.7	0.0957
10	1138.5901	11974	476.4	59070	3.9	0.0951

Figure S38: UV spectrum of compound **4.**

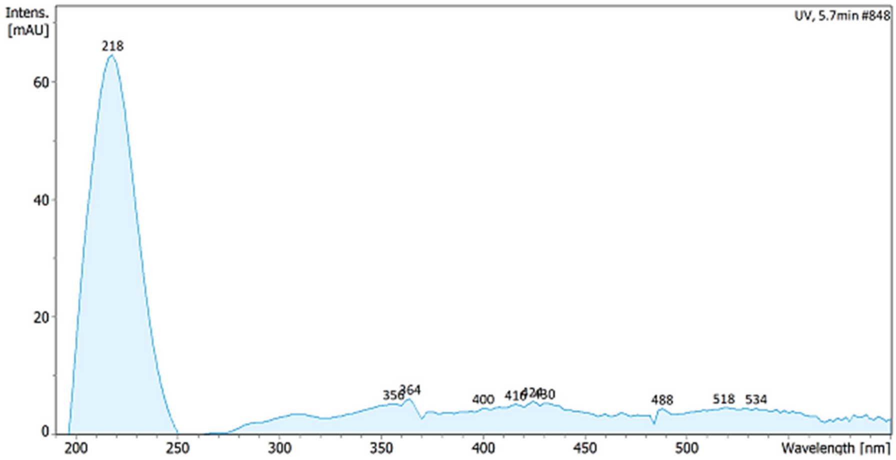

Figure S39: Compound **4**-NMR shift assignment

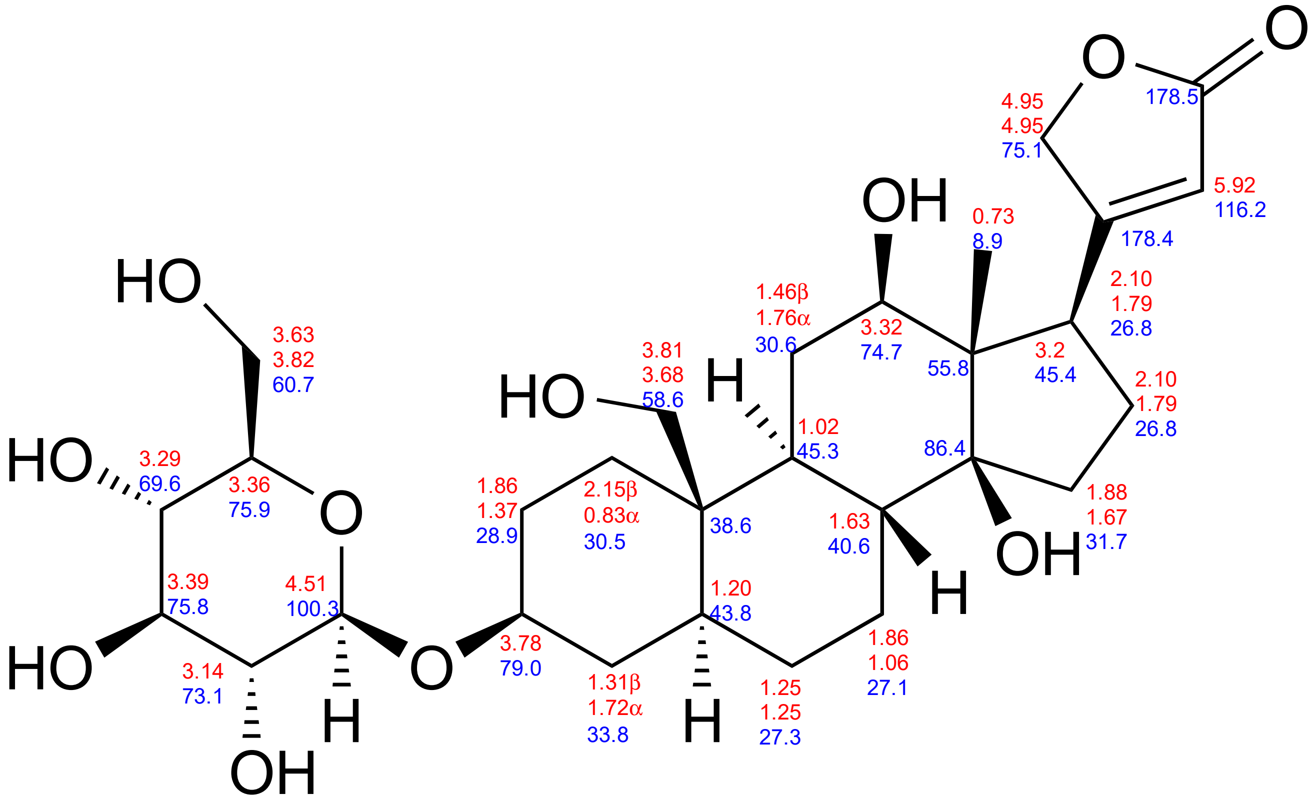

3β-(β-D-glucopyranosyloxy)-12β,14,19-trihydroxy-5α-card-20(22)-enolide

Figure S40. ^{1}H-NMR spectrum (500 MHz, D_2O) of compound **4**.

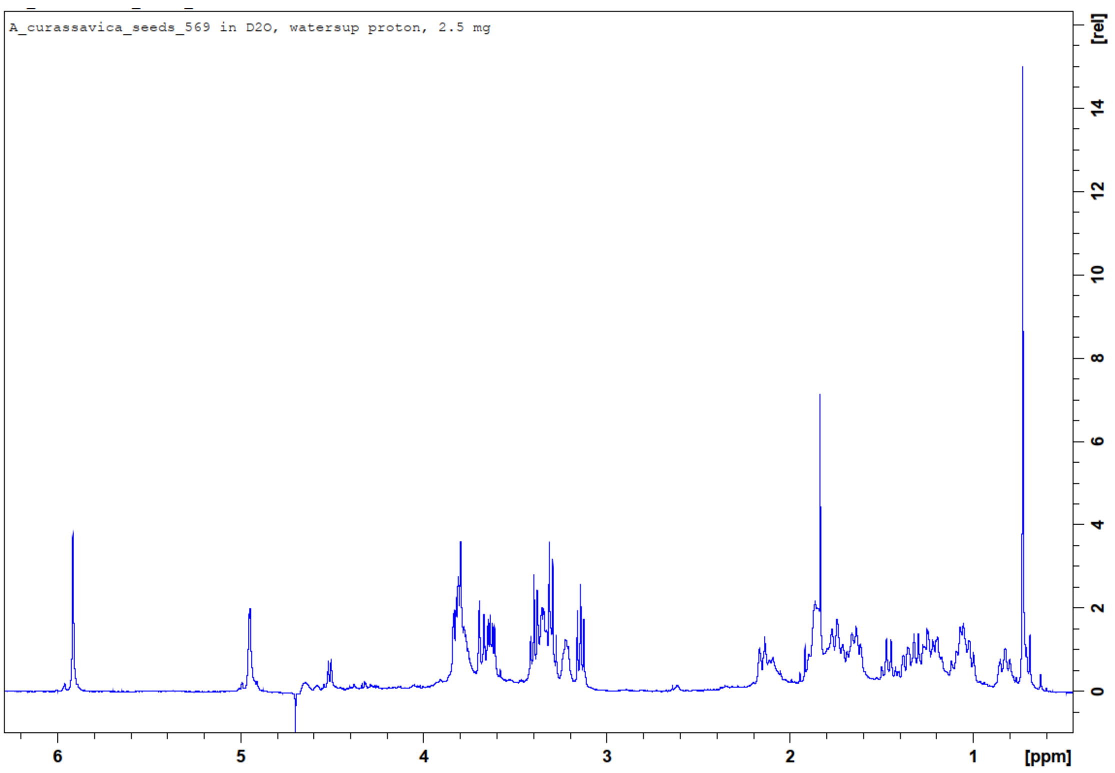

Figure S41. APT spectrum (125 MHz, D_2O) of compound **4**.

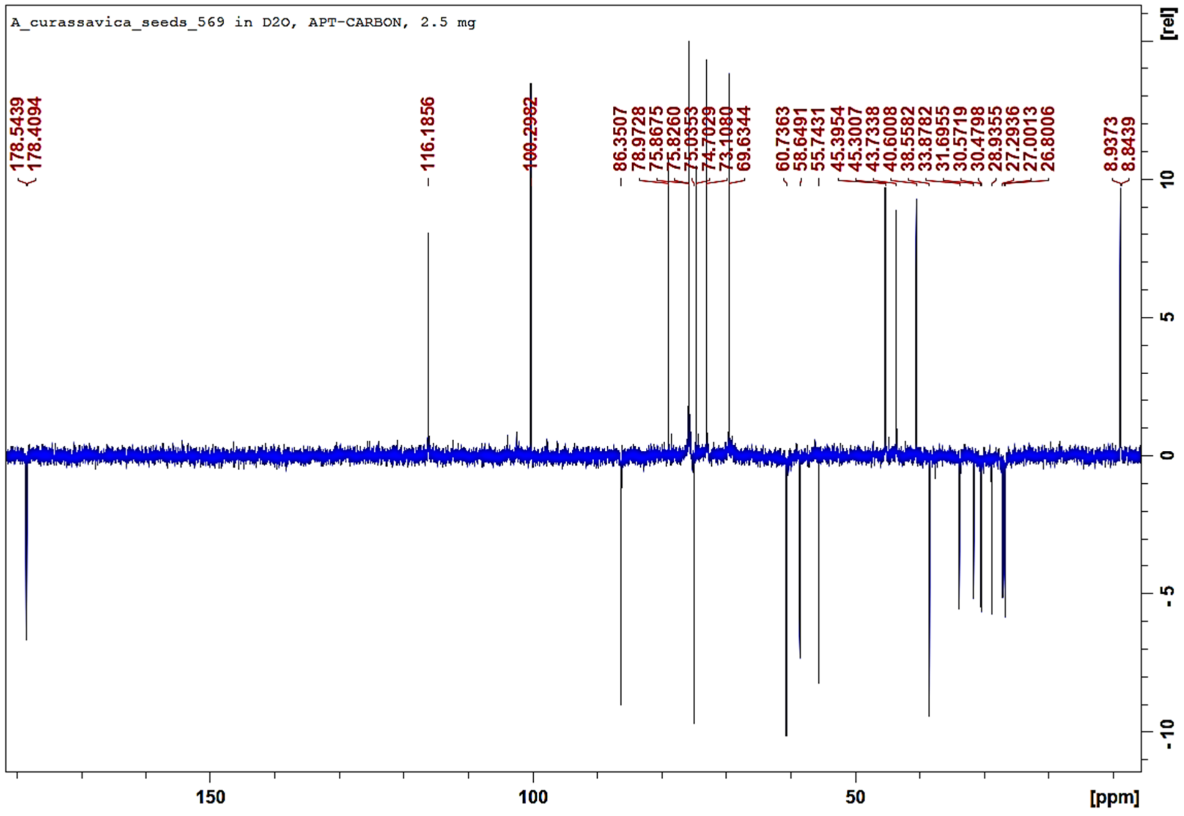

Figure S42. HSQC spectrum (500 MHz, D_2O) of compound **4.**

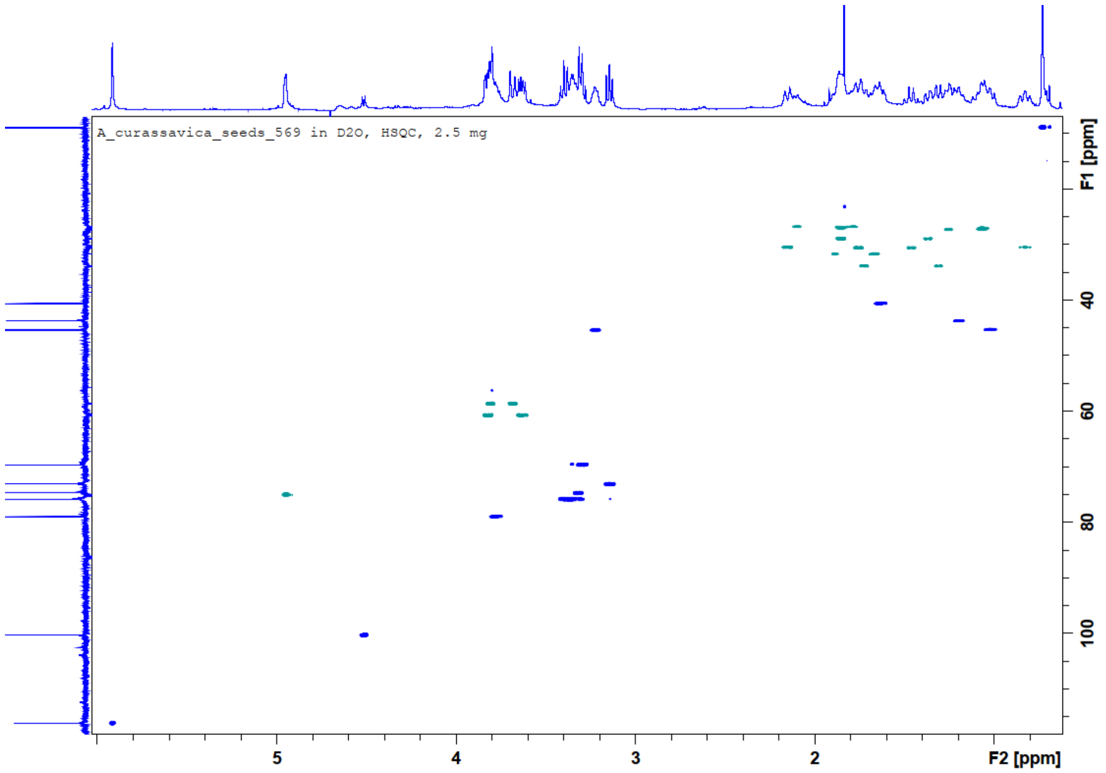

Figure S43. ^1H-^1H COSY spectrum (500 MHz, D_2O) of compound **4.**

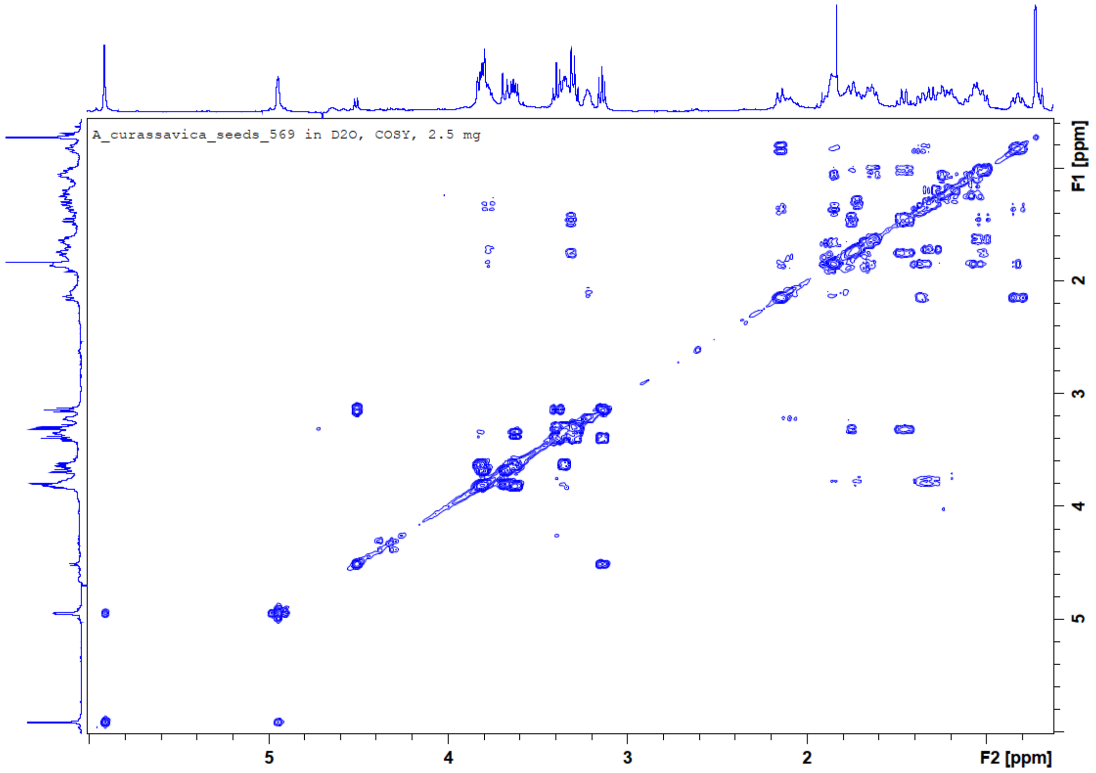

Figure S44. ^1H-^{13}C HMBC spectrum (500 MHz, D_2O) of compound **4.**

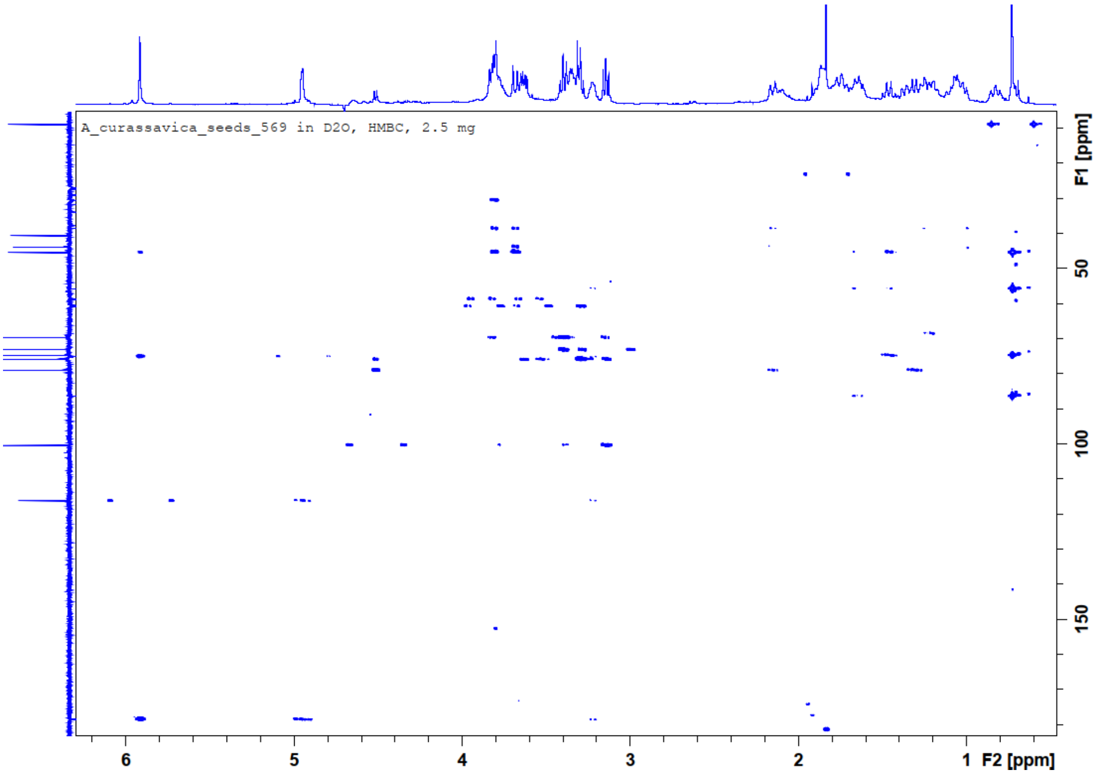

Figure S45. ROESY spectrum (500 MHz, D_2O) of compound **4.**

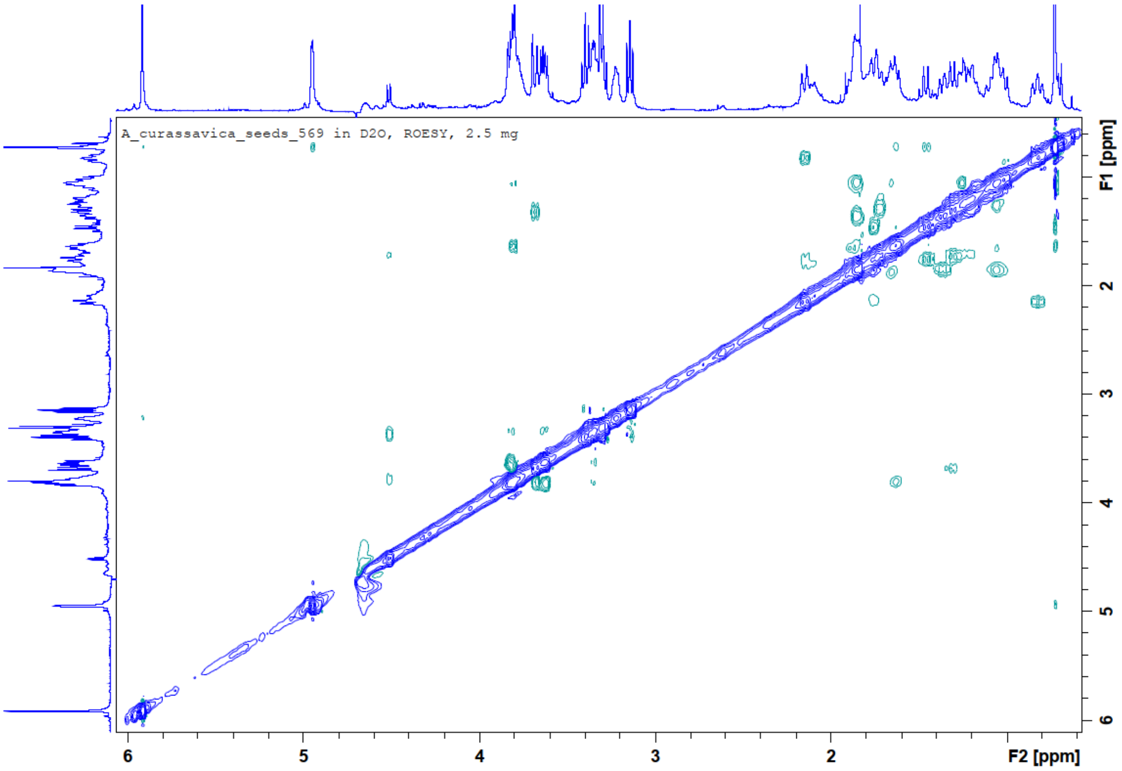

Figure S46. HSQC-TOCSY spectrum (500 MHz, D_2O) of compound **4.**

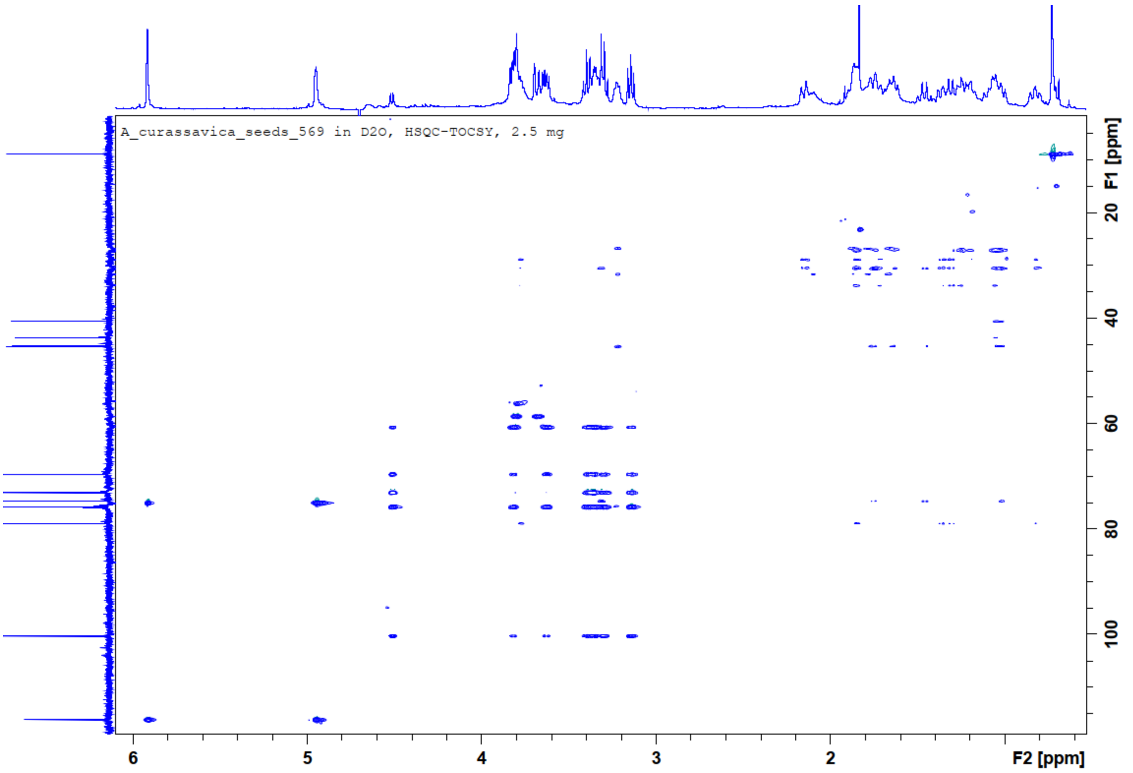

Figure S47. SELTOCSY spectrum (500 MHz, transmitter frequency at 4.514 ppm, D_2O) of compound **4.**

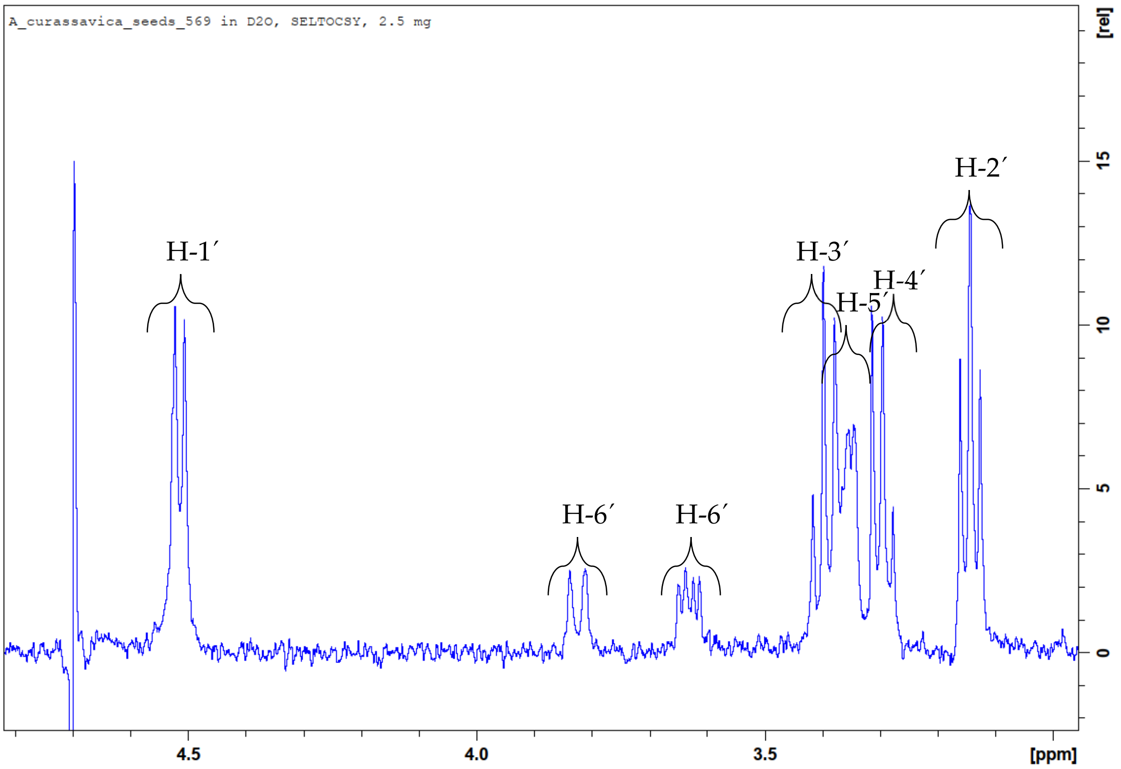

Figure S48. SELNOESY spectrum (500 MHz, transmitter frequency at 3.764 ppm (H-3), D_2O) of compound **4.**

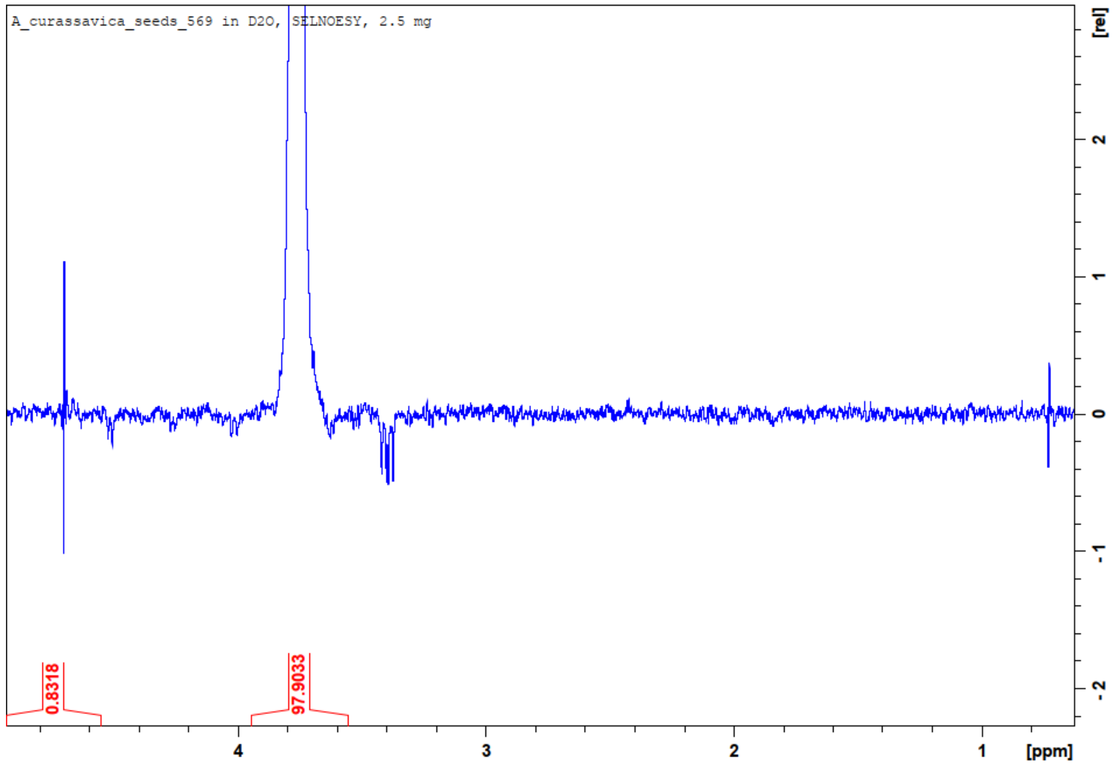

Figure S49. SELNOESY spectrum (500 MHz, transmitter frequency at 3.223 ppm (H-17), D_2O) of compound **4.**

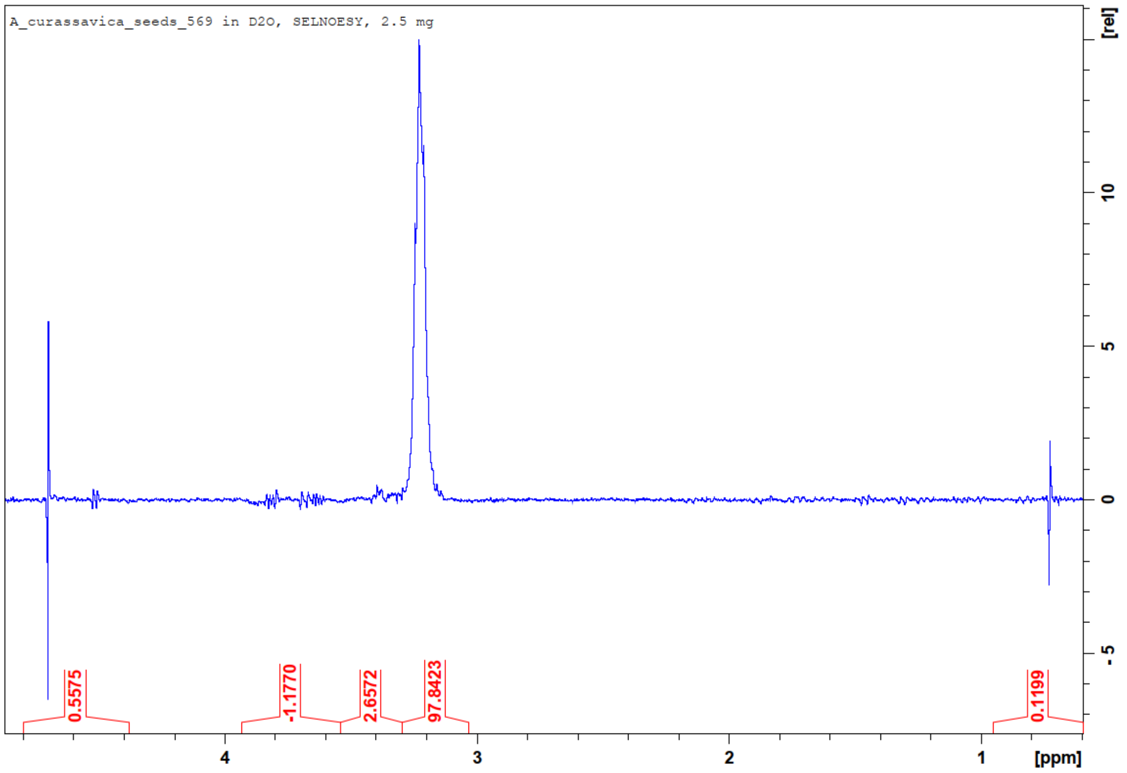

Table S2: Cardenolides quantification (mg/g of seeds)

Compound	Concentration (mg /g of seeds)
**1**	0.014 (+/- 0.0062)
**2**	0.063 (+/- 0.0038)
**3**	0.053 (+/- 0.0029)
**4**	0.004 (+/- 0.0009)
**5**	4.503 (+/- 0.2630)
**6**	2.065 (+/- 0.1930)
**7**	0.733 (+/- 0.0724)
**8**	0.810 (+/- 0.1570)
**9**	0.275 (+/- 0.1040)
**10**	0.457 (+/- 0.3020)

Figure S50: Inhibition curves of *Sus domesticus* Na^+/K^+ ATPase by compounds **1**,**2**, **5**—**10** and ouabain

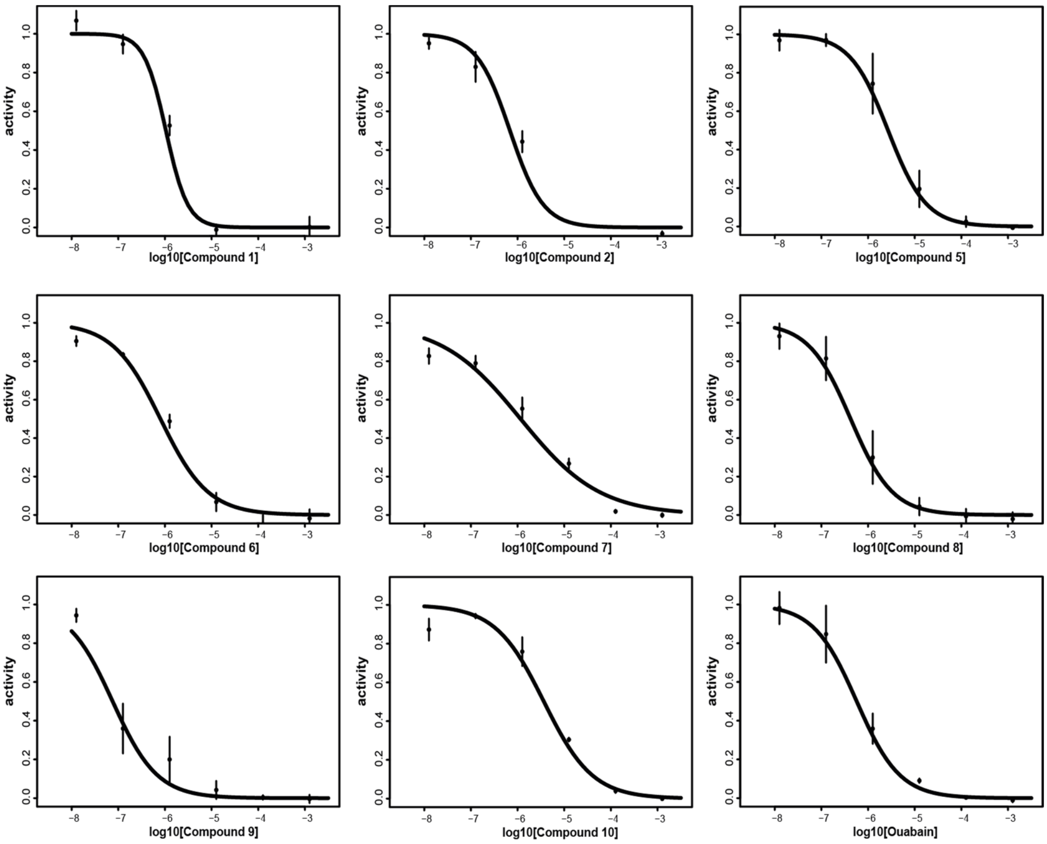

Table S3: IC_{50} values (µM) of compounds **1**,**2**,**5**—**10** and ouabain against porcine Na^+/K^+ ATPase.

**Compound**	**IC_{50} [μM]**
**1**	1.01 x10^{-6} (+/- 1.20 x10^{-8})
**2**	7.42 x10^{-7} (+/- 2.60 x10^{-8})
**5**	2.98 x10^{-6} (+/- 1.69 x10^{-7})
**6**	8.09 x10^{-7} (+/- 8.00 x10^{-9})
**7**	1.20 x10^{-6} (+/- 3.6 x10^{-8})
**8**	4.72 x10^{-7} (+/- 3.00 x10^{-8})
**9**	9.65 x10^{-8} (+/- 6.00 x10^{-9})
**10**	3.67 x10^{-6} (+/- 7.00 x10^{-8})
**Ouabain**	4.48 x10^{-7} (+/- 1.1 x10^{-8})

Table S4: Comparison of IC_{50} values between compounds **1**,**2**,**5**—**10** along with ouabain, p-values adjusted with Bonferroni method (* $p < 0.05$; ** $p < 0.01$, *** $p < 0.001$).

	**1**	**2**	**5**	**6**	**7**	**8**	**9**	**10**	**Ouabain**
**1**		1.000 ns	0.347 ns	1.000 ns	1.000 ns	0.651 ns	**<0.001** ***	**0.046** *	1.000 ns
**2**			**0.035** *	1.000 ns	1.000 ns	1.000 ns	**<0.001** ***	**0.005** **	1.000 ns
**5**				0.083 ns	0.858 ns	**0.001** **	**<0.001** ***	1.000 ns	**0.002** **
**6**					1.000 ns	1.000 ns	**<0.001** ***	**0.011** *	1.000 ns
**7**						0.260 ns	**<0.001** ***	0.119 ns	0.537 ns
**8**							**0.005** **	**<0.001** ***	1.000 ns
**9**								**<0.001** ***	**0.002** **
**10**									**<0.001** ***
